# Point-of-Care CD4 Testing to Inform Selection of Antiretroviral Medications in South African Antenatal Clinics: A Cost-Effectiveness Analysis

**DOI:** 10.1371/journal.pone.0117751

**Published:** 2015-03-10

**Authors:** Andrea L. Ciaranello, Landon Myer, Kathleen Kelly, Sarah Christensen, Kristen Daskilewicz, Katie Doherty, Linda-Gail Bekker, Taige Hou, Robin Wood, Jordan A. Francke, Kara Wools-Kaloustian, Kenneth A. Freedberg, Rochelle P. Walensky

**Affiliations:** 1 Division of Infectious Diseases, Department of Medicine, Massachusetts General Hospital, Boston, Massachusetts, United States of America; 2 General Medicine, Department of Medicine, Massachusetts General Hospital, Boston, Massachusetts, United States of America; 3 Medical Practice Evaluation Center, Department of Medicine, Massachusetts General Hospital, Boston, Massachusetts, United States of America; 4 Division of Infectious Diseases, Brigham and Women’s Hospital, Boston, Massachusetts, United States of America; 5 School of Public Health and Family Medicine, Division of Epidemiology and Biostatistics, University of Cape Town, Cape Town, South Africa; 6 Desmond Tutu HIV Centre, Institute of Infectious Disease and Molecular Medicine, University of Cape Town, Cape Town, South Africa; 7 Division of Infectious Diseases, Department of Medicine, Indiana University School of Medicine, Indianapolis, Illinois, United States of America; 8 Center for AIDS Research, Harvard University, Boston, Massachusetts, United States of America; 9 Department of Health Policy and Management, Harvard School of Public Health, Boston, Massachusetts, United States of America; University of North Carolina School of Medicine, UNITED STATES

## Abstract

**Background:**

Many prevention of mother-to-child HIV transmission (PMTCT) programs currently prioritize antiretroviral therapy (ART) for women with advanced HIV. Point-of-care (POC) CD4 assays may expedite the selection of three-drug ART instead of zidovudine, but are costlier than traditional laboratory assays.

**Methods:**

We used validated models of HIV infection to simulate pregnant, HIV-infected women (mean age 26 years, gestational age 26 weeks) in a general antenatal clinic in South Africa, and their infants. We examined two strategies for CD4 testing after HIV diagnosis: *laboratory* (test rate: 96%, result-return rate: 87%, cost: $14) and *POC* (test rate: 99%, result-return rate: 95%, cost: $26). We modeled South African PMTCT guidelines during the study period (WHO “*Option A*”): antenatal zidovudine (CD4 ≤350/μL) or ART (CD4>350/μL). Outcomes included MTCT risk at weaning (age 6 months), maternal and pediatric life expectancy (LE), maternal and pediatric lifetime healthcare costs (2013 USD), and cost-effectiveness ($/life-year saved).

**Results:**

In the base case, *laboratory* led to projected MTCT risks of 5.7%, undiscounted pediatric LE of 53.2 years, and undiscounted PMTCT plus pediatric lifetime costs of $1,070/infant. *POC* led to lower modeled MTCT risk (5.3%), greater pediatric LE (53.4 years) and lower PMTCT plus pediatric lifetime costs ($1,040/infant). Maternal outcomes following *laboratory* were similar to *POC* (LE: 21.2 years; lifetime costs: $23,860/person). Compared to *laboratory*, *POC* improved clinical outcomes and reduced healthcare costs.

**Conclusions:**

In antenatal clinics implementing *Option A*, the higher initial cost of a one-time POC CD4 assay will be offset by cost-savings from prevention of pediatric HIV infection.

## Introduction

Mother-to-child HIV transmission (MTCT) accounts for 260,000 perinatal HIV infections per year worldwide, over 20,000 of which are in South Africa [1]. The risk of MTCT may exceed 30% without the use of antiretroviral drugs (ARVs), but maternal or infant ARVs during pregnancy and breastfeeding can markedly reduce MTCT [2,3]. Both World Health Organization (WHO) and South African guidelines for the prevention of MTCT (PMTCT) recommend that all pregnant women be tested for HIV in general antenatal care, and that HIV-infected women start lifelong three-drug antiretroviral therapy (ART) if they require treatment for their own HIV infection based on low CD4 count or WHO Stage 3–4 disease [3–6]. Women with less advanced disease may also receive three-drug ART under the newest guidelines, or in many programs, may receive zidovudine monotherapy (AZT) for PMTCT alone [4,7].

CD4 measurement is a more sensitive marker of disease stage than clinical evaluation [8]. In PMTCT programs that prioritize ART for women with advanced HIV infection, rapid three-drug ART initiation following timely return of CD4 results improves maternal health and reduces MTCT risk [3,5,9–12]. Traditional laboratory-based CD4 testing may be associated with delays in specimen transport and return of test results of 2–4 weeks in some urban settings such as Cape Town, South Africa, and up to 4 months in other African PMTCT programs [13,14]. Many women therefore do not receive CD4 results or the CD4-appropriate PMTCT regimen prior to delivery [13,14]. Point-of-care (POC) CD4 assays can eliminate these delays, reduce loss to follow-up (LTFU) between testing and result-return, and increase the proportion of patients initiating three-drug ART [15–18]. Despite their intent to be rapid and suitable for use in a range of settings, POC CD4 assays remain a new technology, with a cost ranging from $10-$26 (2013 USD) in South Africa, 2–3 times the per-test cost of standard, laboratory-based flow cytometry assays [19–22]. Our objective was to project the clinical outcomes, costs, and cost-effectiveness of POC CD4 assays compared to laboratory assays for women identified as HIV-infected in general antenatal clinics (2010–2013) in South Africa.

## Methods

### Ethics and informed consent

This work was approved by the Partners Healthcare Human Subjects Committee, Boston, MA, USA and the University of Cape Town IRB, Cape Town, South Africa. Participants at the study site in South Africa provided written informed consent for this work.

### Analytic overview

In 2012, a point-of-care CD4 assay was introduced and evaluated in the Gugulethu Midwife Obstetric Unit (MOU), an antenatal clinic near Cape Town, South Africa (Appendix) [15]. We used data from this evaluation, with published clinical and cost data, to simulate a cohort of pregnant women identified as HIV-infected in antenatal care and their infants [13,14,21–24]. We linked three validated computer models: 1) a decision analytic model simulating a cohort of women through a single pregnancy and delivery (the “MTCT model”) [25–27]; 2) a Monte Carlo model of HIV disease among postpartum women (the Cost-effectiveness of Preventing AIDS Complications-International or “CEPAC-Adult model”) [28,29]; and 3) a Monte Carlo model of perinatal and postpartum HIV infection among HIV-exposed infants (the “CEPAC-Pediatric model”) [26,30]. Together, these three models simulate each mother-infant pair from the time of presentation to antenatal care (ANC) through the lifetimes of both mother and infant.

We projected short- and long-term clinical and economic impacts for two CD4 testing strategies: flow cytometry performed in a central laboratory (“*laboratory*”), and point-of-care testing performed in the antenatal clinic (“*POC*”). Clinical outcomes included MTCT risk at birth and weaning, pediatric life expectancy from birth, maternal life expectancy from presentation to care, and combined (maternal+pediatric) life expectancy. Economic outcomes, from the healthcare system perspective, included ANC costs, lifetime maternal HIV-related healthcare costs, lifetime pediatric healthcare costs, and 1–5-year maternal and pediatric health care costs (2013 USD). We calculated incremental cost-effectiveness ratios (ICERs) in $/life-year (LY): difference in combined healthcare costs (antenatal+maternal+pediatric costs) between the two strategies divided by difference in combined projected life expectancy (maternal+pediatric life expectancy). For ICERs, all outcomes were discounted at 3%/year [31]. We considered a strategy to be “very cost-effective,” compared to the alternative strategy, if its ICER was <1x South African *per-capita* gross domestic product (GDP: $6,600 in 2013)/LY, “cost-effective” if the ICER was <3x GDP/LY, and “cost-saving” if it led to greater combined life expectancy and lower combined costs [32,33].

### Modeled population and testing/treatment strategies

We projected outcomes for a cohort of HIV-infected, ART-naïve pregnant women and their infants in South Africa (Table 1), following a positive HIV test at the first ANC visit [13]. In *laboratory*, CD4 specimens were shipped to the national laboratory for flow cytometry, with results returned to patients at a second visit three weeks later. In *POC*, CD4 testing and result-return both occurred during the first ANC visit.

**Table 1:** Selected model input parameters for the base-case analysis (See S1 Table for complete list and ranges evaluated in sensitivity analyses).

***Clinical model input parameters***						
***Variable***			***Value***	***Data sources***		
***Baseline maternal cohort characteristics***						
Age (mean (SD), years)			26(5)	[13]		
Mortality during pregnancy			0.26%	[72]		
Proportion with CD4 <350/μL			44%	[13]		
***CD4 assay uptake, result-receipt, and test characteristics***						
	***CD4 tested (of HIV+)***	***Roceiving results (of CD4 tested)***	***CD4 tested and receiving results (of HIV+)***	***Sensitivity (for CD4 ≤350/μL) ^a^***	***Specificity (for CD4 ≤350/μL) ^a^***	***Data Sources***
Laboratory CD4 testing (base case)	96%	87%	83%	100%	100%	[45]
Laboratory CD4 ladling (low-access)	30%	50%	15%	100%	100%	[51]
POC CD4 testing	99%	95%	94%	93%	86%	[18,45]
***Mother-to-child transmission risks: Base-case value***						
***Maternal HIV status***	***PMTCT regimen received***					
***Intrauterine/intrapartum period (one-time risks)***						
	***Antenatal AZT^b^***		***Antenatal three-drug ARV regimen^b^***	***Data sources***		
CD4 <350/μL at conception	0.136		0.033	[61–64,73–76]		
CD4 >350/μL at conception	0.036		0.01	[61–64,73–76]		
***Postnatal period (rate/100 person-years, among infants HIV-uninfected at 4–6 weeks of age)***						
	***Extended infant NVP***		***Antenatal three-drug ARV regimen***	***Data sources***		
CD4 <350/μL	n/a		4.0	[61,62,64,74,76–81]		
CD4 >350/μL	2.7		2.2	[61,62,64,74,76–81]		
***Economic model input parameters***						
***Laboratory and medication costs***			***2013 USD***	***Data sources***		
CD4 assay			Lab: $14/POC: $26	Lab[22]/POC[21]		
CD4 result return (10 min of nurse time to locate and file result)			Lab: $1/POC: $0	Assumption (nurse time x salary)[23]		
Antenatal AZT^b^			$23	[24]		
Antenatal TDF/3TC/EFV^b^			Lab: $36/POC: $40	[24]		
Postnatal maternal ART						
1st-line(TDF/FTC/EFV)			$13	[24]		
2nd-line (AZT/3TC/LPV/r)			$41	[24]		
Pediatric ART (cost varies by age)						
1st-line (ABC/3TC/LPV/r)			$25-$41	[24,82]		
2nd-line (AZT/3TC/NVP)			$6-$15	[24,82]		
Antenatal care						
Routine antenatal care (4 visits)			$200	Assumption		
Delivery costs (healthcare facility)			$60	[83]		
Urgent health care costs: Children						
Care for acute opportunistic infections (per event)						
WHO stage Ill			$1,240	[22,47]		
WHO stage IV			$2,180	[22,47]		
Tuberculosis			$1,650	[22,47]		
Urgent health care costs: Mothers			***# Inpatient days/event***	***# Outpatient visits/event***	***Total cost / event***	
Care for acute opportunistic infections						
WHO stage III-IV disease (range by specific disease)			2.7–3.4	1.3–2.9	$465–875	[22,35,46]
Bacterial Infection			2.8	2.4	$825	[22,35,46]
Mild fungal infection			1.2	2.3	$390	[22,35,46]
Tuberculosis			2.9	2.2	$830	[22,35,46]
Routine HIV care costs (per month): Mothers and children			***# Inpatient days/month***	***# Outpatient visits/month***	***Total cost / month***	
CD4 <500/μL (<35%)			0.03	0,30	$20	[22,35,46]
CD4 351–500/μL (25–35%)			0.06	0.27	$30	[22,35,46]
CD4 201–350/μL (20–25%)			0.08	0.26	$35	[22,35,46]
CD4 101–200/μL (15–20%)			0,22	0.29	$75	[22,35,46]
CD4 51–100/μL (5–15%)			0.22	0.29	$75	[22,35,46]
CD4 μ50/μL (0–5%)			0.56	0.52	$170	[22,35,46]
Terminal care, last month of life: mothers and children			2.39	0.77	$655	[22,35,46]

**SD**: Standard deviation; **ART**: antiretroviral therapy; **PMTCT**: prevention of mother-to-child HIV transmission; **AZT**: azidothymidine (zidovudine); **ARV**: antiretroviral; **NVP**: nevirapine; **ABC**: abacavir; **3TC**: lamivudine; **LPV/r**: lopinavir/ritonavir; **TDF**: tenofovir; **FTC**: emtricitabine; **EFV**: efavirenz; **WHO**: World Health Organization

**a**. Sensitivity and specificity were modeled with regard to true CD4 value of ≤350/μL (sensitivity: assay reports CD4 ≤350/μL when true CD4 is ≤350/μL; specificity: assay reports CD4 >350/μL when true CD4 is >350/μL). To be conservative with regard to the benefit of POC, we assumed in the base case that *laboratory* CD4 had 100% sensitivity and specificity to detect true CD4 ≤350/μL.

**b**. In the base-case analysis, 13 weeks of antentatal AZT for non-ART eligible women are assumed in both strategies, based on median gestational age at booking in South Africa of 26 weeks. For ART-eligible women, 13 weeks of ART are assumed in the *POC* strategy and 3 weeks of AZT and 10 weeks of ART are assumed in the *laboratory* strategy.

**c**. Please see S1 Table for description of assumptions of outpatient healthcare resource utilization.

**Fig 1:**
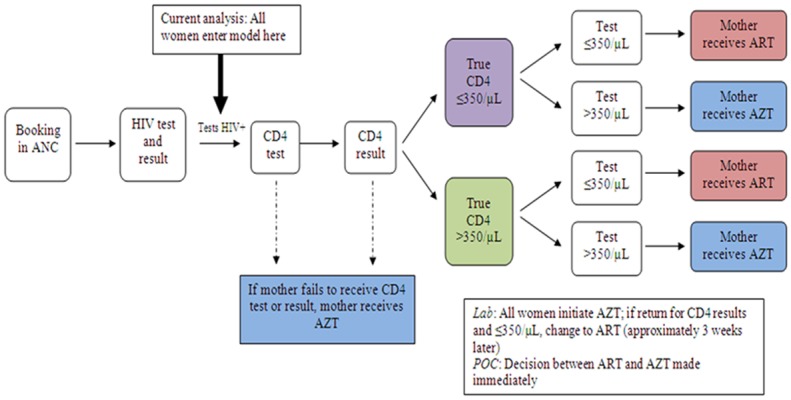
Model structure. This figure shows the modeled sequence of events during antenatal care that determine a mother’s prescribed PMTCT drug regimen. During the first visit, all women receive an HIV test and HIV test results. In the current analysis, all women who are HIV-infected are assumed to have positive HIV test results and enter the MTCT model, at which point they are assigned a probability of undergoing a CD4 test and, if tested, a probability of receiving their CD4 test results. Women are also modeled to be eligible for ART (true CD4 ≤350/μL) or non-eligible for ART (true CD4 >350/μL) based on 2010 WHO guidelines. The sensitivity and specificity of the CD4 assays are reflected in assigned probabilities that the CD4 test will indicate women to be eligible or non-eligible for ART. The observed CD4 results then determine whether women receive AZT or ART for PMTCT. Transmission probabilities and maternal outcomes depend on true CD4 count and PMTCT regimen received. **Abbreviations: ANC**: antenatal care; **POC**: point-of-care testing; **ART**: three-drug antiretroviral therapy; **AZT**: zidovudine.

We simulated South African PMTCT guidelines at the time of the study, which reflected WHO *Option A* (lifelong maternal three-drug ART if CD4 ≤350/μL or WHO Stage 3–4 disease; maternal zidovudine (AZT) in pregnancy, then daily infant nevirapine (NVP) throughout breastfeeding if CD4 >350/μL; Fig. 1) [3,5]. In the antenatal period, we therefore modeled provision of AZT to women who were awaiting CD4 results (*laboratory*), who never received CD4 results (either strategy), or who received results indicating CD4>350/μL (either strategy). We modeled provision of antenatal three-drug ART to women who received results indicating CD4 ≤350/μL or who had evidence of WHO Stage 3–4 disease (either strategy). As a result, the model permits women with CD4 <350/μL to “incorrectly” receive AZT instead of ART, and thus have higher MTCT risks (Table 1), in order to incorporate the critical role of CD4 testing in selecting antenatal ARV regimens.

In the absence of data on infant feeding practices under recent South African guidelines, we modeled six months of breastfeeding for all infants [6]. After delivery, modeled women in both strategies linked to postnatal care, including routine laboratory-based CD4 monitoring. Women with initial or current CD4 ≤350/μL continued lifelong ART, and those with initial and current CD4 >350/μL stopped maternal AZT and provided daily nevirapine syrup to their infants until weaning [3,5].

To isolate the impact of the CD4 testing strategies, in the base case, we varied only CD4 testing rates, CD4 result-return rates, and CD4 assay costs. We otherwise assumed guideline-concordant care based on receipt of CD4 results: all women were accurately identified as HIV-infected, all mothers and infants adhered to prescribed PMTCT regimens, and all mothers and infants linked to postnatal HIV-related care, received ART if eligible after delivery, and were retained in care. To be conservative with regard to the benefit of *POC*, we assumed in the base case that *laboratory* CD4 had 100% sensitivity and specificity to detect true CD4 ≤350/μL, and that *POC* had 93% sensitivity and 86% specificity to detect true CD4 ≤350/μL (Table 1) [18]. We varied all of these assumptions in sensitivity analyses.

### Model structure

We linked three computer models to simulate mother-infant pairs through pregnancy, breastfeeding, and the lifetimes of both mothers and infants (S1 Appendix; S1–S2 Figs.) [25–29,34]. At the time of presentation to ANC, mother-infant pairs enter the MTCT model, in which they face probabilities of key clinical events during pregnancy and delivery. MTCT model outcomes are assessed after delivery, and include maternal and infant vital status, infant HIV infection, and costs accrued during pregnancy and delivery. From delivery through death, clinical and economic outcomes are simulated for mothers in the CEPAC-Adult model and for infants in the CEPAC-Pediatric model. In these models, individuals are subject to monthly risks of clinical events, including opportunistic infections, response to ART, medication toxicities, and mortality, and the costs associated with these events (Appendix).

### Model input parameters

#### Cohort characteristics, disease progression, and ART

We simulated the cohort of women seeking care at the Gugulethu MOU, with median age of 26 years and median gestational age at first visit of 26 weeks (Table 1, S1 Table) [13]. Monthly risks for opportunistic infections (OIs) and HIV-related death in the absence of ART were from Cape Town (adults) and from the International Epidemiologic Database for the Evaluation of AIDS (IeDEA; children) [30,35,36]. First-line ART was tenofovir/emtricitabine/efavirenz (TDF/FTC/EFV) for women and abacavir/lamivudine/lopinavir/ritonavir (ABC/3TC/LPV/r) for HIV-infected children [3,37–39]. Further details of ART initiation, CD4 and RNA responses to ART, and switching to second-line ART regimens are provided in the Appendix [40–44].

#### MTCT risks

Modeled MTCT risks during pregnancy and breastfeeding, which substantially impact projected pediatric life expectancy, were the average values from published clinical studies in African breastfeeding populations, stratified by maternal CD4 count and ARV regimen received (Table 1, S1 Table, S1 Appendix) [26]. In sensitivity analyses, we also examined the impact of the highest and lowest published transmission risks for each regimen and CD4 stratum (S1 Appendix).

#### Effectiveness of CD4 testing strategies

We defined two key parameters for each CD4 testing strategy: the proportion of HIV-infected women undergoing CD4 testing, and the proportion of CD4-tested women receiving CD4 results and initiating three-drug ART if CD4 ≤350/μL (result-return, Table 1; result-return rates below 100% reflect the proportion of women lost to follow-up before receiving CD4 results). For the *laboratory* strategy, data for testing (96%) and result-return (87%) were from the Cape Town MOU [45]. For the *POC* testing strategy, data for testing (99%) and result-return (95%) were from the pilot study of POC CD4 measurement at the MOU [45]. Based on MOU data, we modeled a 3-week interval between CD4 testing and CD4 result-return for the *laboratory* strategy [14].

#### Costs

POC CD4 assay costs (base case: $26) were derived according to Larson *et al*., substituting healthcare worker time observations from the MOU and local salary data in place of the Larson estimates (Table 1, S1 Table) [21]. Laboratory-based CD4 assay costs (base case: $14) were from published data [22]. During pregnancy, we included the costs of routine antenatal care and delivery (Appendix). After delivery, we included maternal and pediatric costs for routine HIV-related healthcare, acute care for opportunistic infections, ART, laboratory monitoring, and care in the final month of life (Appendix) [22,35,46,47]. All costs were in 2013 US dollars.

### Model validation and sensitivity analyses

In previous work, we validated model-projected MTCT risk, pediatric survival, pediatric HIV-free survival, and maternal postpartum OI rates against published data, and we reported extensive sensitivity analyses on clinical, cost, and access-to-care parameters [25–28]. For this analysis, we examined additional variations in test sensitivity, specificity, testing rates, and result-return rates for the *POC* strategy, as well as antenatal and postnatal loss to follow-up (LTFU) rates, breastfeeding duration, healthcare and medication costs, MTCT risks, and the discount rate for both *POC* and *laboratory* strategies (S1 Table). We also examined both decreased POC CD4 costs, reflecting new POC assays in development, and increased POC CD4 costs, to incorporate possible costs not captured in the base-case estimate, for example: additional healthcare worker time to process CD4 specimens, undergo training, or conduct quality control activities; or reduction in staff capacity to perform other patient-related activities [21,48–50]. Finally, we conducted multiway sensitivity analyses, varying *POC* assay cost, sensitivity, testing rates, and result return rates simultaneously.

### Low laboratory access scenario

In many settings, access to laboratory-based CD4 testing is more limited than in Cape Town, an urban area close to central laboratory facilities. We therefore examined a second, “low laboratory access scenario,” in which *POC* CD4 testing was introduced into a setting with a *laboratory* test rate (30%) and result-return rate (50%) based on UNAIDS data for low/middle-income countries [51].

### Budget impact analysis

To inform short-term budgets, we projected not only lifetime outcomes, but also outcomes over a 5-year horizon. Outcomes included ANC costs, pediatric costs, total costs, and yearly pediatric survival rates for both *laboratory* and *POC* strategies. Because variations in access to laboratory-based CD4 testing had the greatest impact on lifetime cost projections, we repeated the budget impact analysis in the “low laboratory access” scenario.

## Results

### Base-case results

*Laboratory CD4 strategy*. *Laboratory* resulted in a 4.2% MTCT risk at birth and 5.7% MTCT risk at 6 months (Table 2). This strategy led to a pediatric life expectancy of 53.2 years (23.50 years discounted) and a maternal life expectancy of 21.2 years (14.8 years discounted), for a combined life expectancy of 74.3 years (38.3 years discounted). ANC costs were $310/mother, lifetime pediatric costs were $760/infant ($520 discounted), and lifetime maternal costs were $23,860/person ($15,440 discounted), for a combined cost of $24,930/mother-infant pair ($16,270 discounted).

**Table 2:** Base-case results: projected outcomes for point-of-care and laboratory-based CD4 testing in antenatal care in South Africa.

CD4 testing strategy	MTCT at birth	MTCT at 6 months	ANC costs	Pediatric ^a^		Maternal ^a^		Maternal + Pediatric ^a^		Incremental cost-effectiveness ratio
				Life expectancy (years)	Lifetime cost (US$)	Life expectancy (years)	Lifetime cost (US$)	Life expectancy (years)	Lifetime cost (US$)	
**Base-case analysis**										
*POC*	3.8%	5.3%	325	53.35 (23.56)	710 (480)	21.15 (14.78)	23,860 (15,440)	74.50 (38.34)	24,900 (16,250)	
*Laboratory*	4.2%	5.7%	310	53.18 (23.50)	760 (520)	21.15 (14.78)	23,860 (15,440)	74.33 (38.28)	24,930 (16,270)	Dominated^b^
**Low laboratory access scenario** ^c^										
Low-access laboratory*	7.3%	8.7%	295	51.93 (23.05)	1,180 (810)	21.14 (14.77)	23,850 (15,430)	73.07 (37.82)	25,330 (16,540)	Dominated^b^

**MTCT**: mother-to-child transmission; **ANC**: antenatal care (costs accrued during pregnancy and delivery); **POC**: point-of-care CD4 testing strategy.

*compared to *POC*

**a**. Undiscounted life expectancies and costs are shown without parentheses. Life expectancy and cost projections were also discounted at a rate of 3% per year, shown in parentheses. ANC costs were not discounted, because they accrued in the first year of the simulation. All costs are in 2013 USD. Projections are shown for a cohort of HIV-infected mothers after delivery, and for a cohort of their infants from birth (most of whom are HIV-uninfected).

**b**. *Laboratory* testing strategies were dominated, meaning that they were more expensive (higher total ANC+maternal+pediatric costs) and less effective (lower total maternal+pediatric life expectancy) than the *POC* testing strategy.

**c**. *POC* results remain unchanged in the low laboratory access scenario.

#### Point-of-care CD4 strategy

*POC* resulted in a 3.8% MTCT risk at birth and a 5.3% MTCT risk at 6 months. This strategy led to a pediatric life expectancy of 53.4 years (23.6 years discounted) and a maternal life expectancy of 21.2 years (14.8 years discounted), for a combined life expectancy of 74.5 years (38.3 years discounted). ANC costs were $325/mother, lifetime pediatric costs were $710/infant ($480 discounted), and lifetime maternal costs were $23,860/person ($15,440 discounted), for a combined cost of $24,900/mother-infant pair ($16,250 discounted). Over a lifetime horizon for mother and infants, *POC* was cost saving compared to *laboratory*, with greater combined maternal and pediatric life expectancy and lower combined costs (Table 2).

### Sensitivity analyses

#### POC sensitivity and specificity

**Fig 2:**
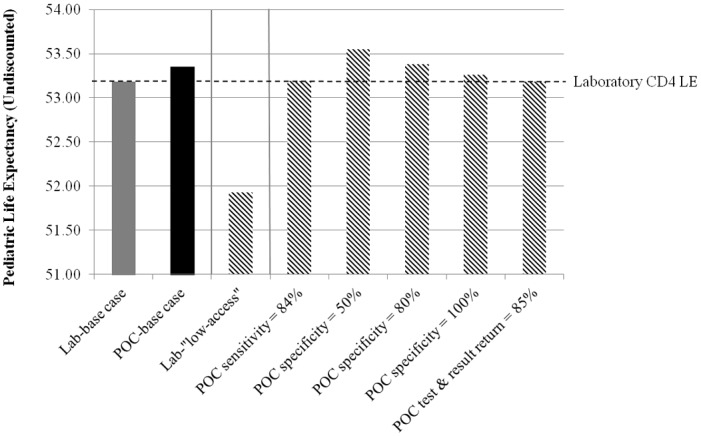
Univariate sensitivity analyses: Pediatric life expectancy. Undiscounted pediatric life expectancies for *laboratory* and *POC* testing are shown (maternal life expectancies do not differ substantially by testing strategy, and so are excluded from the figure). *POC* “test and result return” is defined as the product of (proportion of HIV-identified women undergoing CD4 testing) * (proportion of CD4-tested women receiving CD4 results). For *POC* sensitivity and *POC* “test and result return,” life expectancies are shown at the threshold values at which *POC* testing no longer results in a higher life expectancy compared to *laboratory* testing. For *POC* specificity, life expectancy increases as specificity decreases, so no such threshold exists; results are shown at 50%, 80%, and 100%, as examples. The horizontal dotted line shows the undiscounted pediatric life expectancy under the base case laboratory conditions. Left panel: base case; middle panel: low laboratory access scenario; right panel: sensitivity analyses on *POC* parameters. **Abbreviations**: *POC*: point-of-care testing.

Holding test specificity at the base-case value of 86%, *POC* CD4 testing remained cost-saving (greater life expectancy and lower costs) compared to *laboratory* unless *POC* test sensitivity was ≤89%. *POC* resulted in a higher combined life expectancy compared to *laboratory* unless *POC* test sensitivity was ≤84% (Fig. 2: right panel). Holding test sensitivity at the base-case value of 93%, *POC* life expectancy increased compared to the base-case as the specificity of the POC assay decreased (i.e., more women with high CD4 were “incorrectly” assigned to ART than to AZT), and *POC* life expectancy never fell below *laboratory* life expectancy even at *POC* specificity of 100%. *POC* remained cost-saving (greater life expectancy and lower costs) compared to *laboratory* at all POC assay specificities.

#### POC testing and result return

*POC* remained cost-saving (greater life expectancy and lower costs) compared to *laboratory* unless the proportion of women tested and receiving *POC* assay results was ≤89%. *POC* led to a greater life expectancy compared to *laboratory* unless the proportion of women tested and receiving *POC* assay results was ≤85% (Fig. 2: right panel).

#### POC assay cost

Compared to *laboratory* testing, *POC* remained cost-saving unless the *POC* assay cost ≥$51.

#### Other univariate sensitivity analyses

Results of sensitivity analyses on antenatal or postnatal loss to follow-up for mothers or infants, breastfeeding duration, healthcare and medication costs, a range of MTCT risks (including 6-week risk of 3.0% observed in a recent nationally representative sample [52]), and the discount rate are shown in S3 Table. Although projected MTCT risks, life expectancies, and costs differed as expected from the base case, the comparison between *POC* and *laboratory* was not sensitive to changes in these parameters, assuming they were varied similarly for both strategies.

#### Multivariate sensitivity analyses

**Fig 3:**
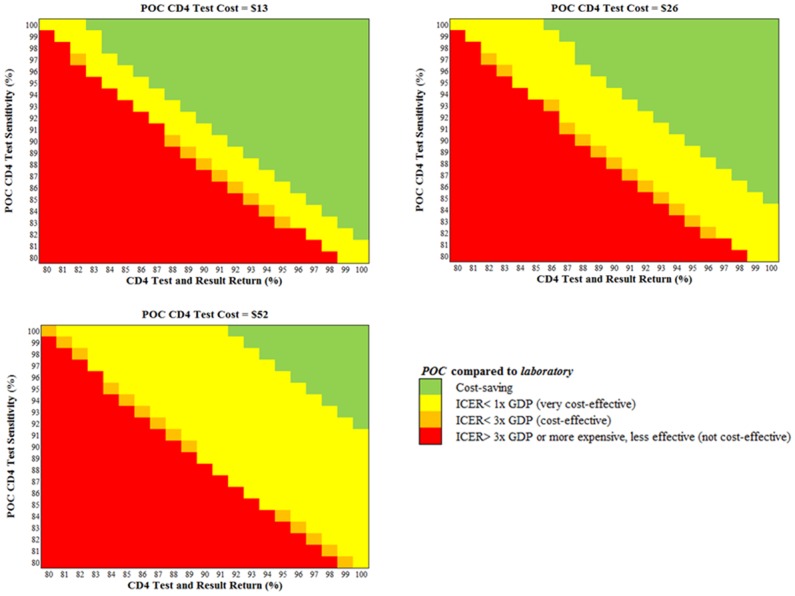
Multivariate sensitivity analyses: Cost-effectiveness of POC CD4 testing compared to laboratory testing. The cost-effectiveness of *POC* CD4 testing compared to *laboratory* testing is shown for key combinations of *POC* CD4 assay cost, *POC* assay sensitivity, and *POC* CD4 test and result return rates, defined as the product of (proportion of HIV-identified women undergoing CD4 testing) * (proportion of CD4-tested women receiving CD4 results). **Abbreviations**: POC: point-of-care testing.

When *POC* sensitivity, *POC* testing and *POC* result-return were high, *POC* was cost-saving at all *POC* assay costs from $13–52 (Fig. 3, upper right corners). Conversely, at very low *POC* sensitivity, testing, and result-return rates, *POC* became more expensive and less effective than *laboratory* (Fig. 3, lower left corners). As *POC* assay cost increased, fewer combinations of sensitivity, testing rates, and result-return rates allowed *POC* to be cost-saving; however, *POC* was cost-effective or very cost-effective in many of these scenarios (Fig. 3, band from upper left to lower right corners).

### Low laboratory access scenario

With lower rates of testing and result-return reflecting UNAIDS data [51], *laboratory* resulted in MTCT risks at birth and 6 months of 7.3% and 8.7%, as well as a pediatric life expectancy of 51.9 years (23.1 years discounted), a maternal life expectancy of 21.1 years (14.8 years discounted), and a combined life expectancy of 73.1 years (37.8 years discounted; Table 2 and Fig. 2: middle panel). Projected *laboratory* ANC costs were $295/mother, lifetime pediatric costs were $1,180/infant ($810/infant discounted), and lifetime maternal costs were $23,850/person ($15,430 discounted), for a combined cost of $25,330/mother-infant pair ($16,540 discounted, Table 2). Cost savings from *POC* relative to *laboratory* were greater in this low laboratory access scenario than in the base case.

### Budget impact analysis

**Fig 4:**
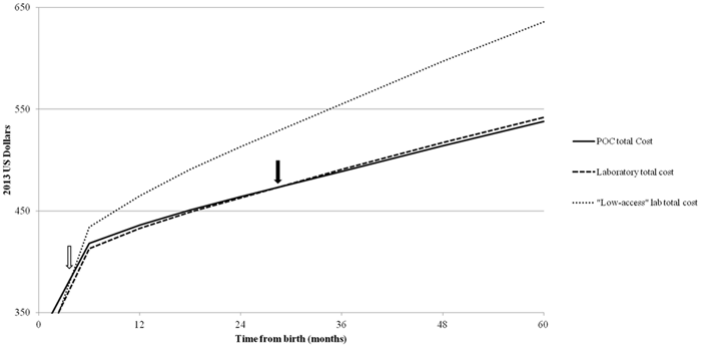
Budget impact analysis. Antenatal and pediatric care costs are shown for the first five years after birth. We include the *POC* and *laboratory* base case strategies, as well as the low laboratory access scenario. The arrows indicate the time points at which the upfront higher costs of *POC* testing are recovered due to savings in pediatric care costs. The open arrow indicates that *POC* becomes cost-saving compared to “low-access” *laboratory* testing within six months of delivery; the closed arrow indicates that *POC* becomes cost-saving compared to the base-case *laboratory* testing strategy within 36 months after delivery. Costs over the first five years after birth are further detailed in S4 Table. Maternal costs were nearly equivalent for both strategies, and are not shown. The sharp inflection point in costs at 6 months after delivery represents the cessation of breastfeeding the associated costs for infant nevirapine for postnatal MTCT prophylaxis. **Abbreviations**: POC: point-of-care testing; ANC: antenatal.

In the base-case analysis, the higher upfront costs of *POC* were offset within 36 months after birth compared to *laboratory*, when both strategies reached costs of $490/mother-infant pair (Fig. 4, solid arrow). In the low laboratory access scenario, the upfront costs of *POC* were offset within 6 months after birth compared to *laboratory* (Fig. 4, open arrow). In both the base-case and low laboratory access scenarios, pediatric survival was slightly greater with *POC* than with *laboratory* at all time points (S4 Table).

## Discussion

In antenatal care, use of laboratory-based CD4 assays can lead to delayed result-return, loss to follow-up, and missed opportunities for PMTCT. In South African PMTCT programs prioritizing ART for women with advanced HIV infection (*Option A*), our model-based analyses suggested that any improvement in testing rates or result-return rates will more than offset the cost of point-of-care CD4 assays, and that the benefits of *POC* testing will be even greater in settings where access to laboratory-based CD4 testing is limited. Projected cost savings were due to the greater number of pediatric HIV infections averted with *POC*; savings occurred within 36 months after delivery in regions with high access to *laboratory* CD4 testing and within 6 months in regions with lower access to *laboratory* CD4 testing [51], and persisted throughout the lifetimes of mother-infant pairs. Although *POC* CD4 assays are a newer, more expensive technology, they may provide the greatest clinical and economic benefits in settings with the most limited healthcare resources.

Our analysis was based on South African PMTCT guidelines during the study period, which followed the 2010 WHO-recommended *Option A* strategy with an ART-eligibility CD4 threshold of 350/μL. As of 2013, South African guidelines now recommend that programs transition to WHO’s *Option B* (three-drug ART for all HIV-infected women during pregnancy and breastfeeding regardless of CD4, with cessation of ART after weaning for women with high initial CD4). Based on a programmatic goal to harmonize treatment for pregnant and non-pregnant patients, many other countries are also planning to implement *Option B*; in addition, many programs are moving toward an ART-eligibility CD4 threshold for non-pregnant patients of 500/μL. As these transitions occur, the role of *POC* CD4 testing in antenatal care will evolve from the scenarios examined in our analysis. For example, with *Option B*, CD4 testing will be necessary to inform decisions about lifelong ART (women with lower pre-ART CD4) versus discontinuation of ART after weaning (women with higher pre-ART CD4), however, rapid POC CD4 result-return in ANC will not impact the choice of antenatal ART versus zidovudine [4]. If programs implement *Option B+* (lifelong ART regardless of initial CD4) [53,54], *POC* CD4 assays may play a role in monitoring ART and switching to second-line ART [55–58].

The current analysis did not examine these different roles for POC CD4 assays in *Options B/B+*. At present, the majority of HIV-infected pregnant women in Africa are still treated under *Option A* [59]. Although a shift to *Option B/B+* is planned in many countries, this will likely require months or years to complete [4,7,59]. Based on available MTCT data and PMTCT guidelines during the study period, we also modeled a CD4 threshold of 350/μL to determine use of AZT versus ART [2,5,60]. The transitions away from *Option A* and ART initiation CD4 thresholds of ≤350/μL will likely occur most gradually in remote and more resource-limited settings, which also have the poorest access to laboratory-based CD4 testing [51]. While the planned transition to *Options B/B+* is ongoing, the majority of HIV-infected, pregnant women in Africa are still receiving interventions based on CD4 counts [59]. In these programs, the rapid result-return permitted by *POC* CD4 testing could substantially improve linkage to HIV care and ART initiation for pregnant women, leading not only to clinical benefits, but also to cost savings.

Notably, we found that with *Option A*, reducing modeled *POC* test specificity improved the projected clinical and economic benefits of *POC* testing. This occurred because, using the average of published MTCT risks for each regimen (Appendix), we modeled lower intrauterine/intrapartum transmission risks with maternal three-drug ART than with AZT [61–64]. Reductions in *POC* specificity led more women with high CD4 to start three-drug ART “incorrectly,” reducing MTCT risks and increasing pediatric life expectancy. Although this was not the focus of our analysis, this finding provides additional support for the universal initiation of ART during pregnancy [3,6]. Our modeled MTCT risks were not based on a direct comparison of the *Option A* and *Option B* regimens in women with high CD4, however; a randomized trial comparing these two regimens is in progress [65]. Additional impacts of *Option B* compared to *Option A*, such as effects on maternal disease progression, prevention of HIV transmission to sexual partners, and reduced risk of maternal or infant tuberculosis, were beyond the scope of this analysis [25,66–68].

There are several limitations to this analysis. First, models necessarily simplify complex clinical and operational processes. Although our three linked models with differing structures did not permit probabilistic sensitivity analyses or generation of 95% confidence intervals, we followed international guidance and conducted extensive univariate and multivariate sensitivity analyses to examine which assumptions and data inputs led to changes in policy conclusions [69]. Second, we assumed a sensitivity and specificity of 100% for *laboratory* CD4 testing, as the gold standard. We chose this simplifying assumption to be conservative with regard to the benefits of *POC* CD4 testing, as well as to accurately map modeled CD4 strata to those in the PMTCT trials providing input data, which used laboratory CD4 measurements. Third, our analyses may exclude unforeseen impacts for women who receive HIV test results, learn CD4 results, and initiate ART at the first ANC visit. Although pilot data suggest high levels of acceptance for *POC* CD4 result-return and rapid ART initiation [14], if this process leads women to feel overwhelmed, and thus to maintain poor adherence and retention in care at later time points, the benefits of POC CD4 testing may be attenuated [70]. Finally, POC CD4 assays permit initiation of ART several weeks earlier than laboratory-based testing. Receipt of three weeks of ART in place of AZT will likely have minimal impact on maternal health, but may substantially reduce MTCT risk, especially late in gestation [11,12,71]. To include this effect, the model would require MTCT risks stratified by both duration of AZT and ART use and the gestational age at which each regimen is initiated. We were unable to identify such data, so we examined only improvements in testing and result-return rates related to *POC*. Because we did include the additional costs of three weeks of ART, however, this was a deliberately conservative assumption; including the MTCT reduction from three additional weeks of ART would likely show *POC* to be even more effective, with even greater cost-savings, compared to *laboratory*.

In conclusion, although additional funding will be needed to implement POC CD4 testing in the short-term, we find that POC CD4 testing will improve clinical outcomes and will save money within 1–3 years of delivery, compared to laboratory-based CD4 testing, in a range of settings. POC CD4 testing should be implemented in PMTCT programs that prioritize ART for women with advanced HIV infection.

## Supporting Information

S1 Appendix Point-of-care CD4 Testing to Inform Selection of Antiretroviral Medications in South African Antenatal Clinics: a Cost-effectiveness Analysis. (DOCX)

S1 Fig PMTCT “cascade;” schematic representation of the MTCT model (adapted with permission from Ciaranello *et al*, *PLoS ONE*, 2011; 6(6)). The MTCT model is a decision tree, coded in TreeAgePro software. Pregnant women enter the model at conception. Five possible PMTCT strategies are shown at the decision node, indicated by a square. This analysis examined only Option A. Circles indicate chance nodes, at which events occur based on probabilities derived from published literature. Triangles indicate terminal nodes, representing the clinical outcome of any single pathway through the model. Brackets reflect that the subsequent events emerging to the right of the bracket may follow any of the prior chance nodes included to the left of the bracket. At each chance node, the probabilities of all subsequent modeled events may depend on the PMTCT strategy being simulated and on the prior events leading to that node. For each modeled PMTCT strategy, the series of events shown in the figure may occur. For example, HIV-infected women may be ART-eligible (CD4≤350/μL or WHO Stage 3–4 disease) or non-ART-eligible; ART-eligibility may be identified by CD4 testing, identified by clinical evaluation, or not identified. All women may access ANC, undergo HIV testing in ANC, and receive HIV test results, or may fail to access these steps in the cascade. If identified as HIV-infected, women may be offered ARVs for PMTCT according to the PMTCT strategy being simulated, as well as ART if identified as ART-eligible (not shown). In the base case, women were assumed to receive all ANC services; service uptake was varied in sensitivity analyses. Probabilities for surviving pregnancy depend on receipt of ART; if maternal death occurs, infant death also occurs. Women who survive pregnancy may deliver at a healthcare facility or at home; if they deliver in a healthcare facility, they may access HIV testing (if previous status was unknown or negative), and if identified as HIV-infected at that time, may receive sdNVP in labor (sdNVP was excluded for this analysis). All women surviving pregnancy then experience probabilities of live birth and HIV infection in their infants, depending on PMTCT regimen received. Finally, women may link or fail to link to postnatal HIV-related care for themselves. At the end (far right) of any given path through the model, there are two sets of outcomes: infant outcomes and maternal outcomes. Infant outcomes include HIV infection status (infected or uninfected at birth, shown), risk of postnatal HIV infection if uninfected at birth, life expectancy, and per-person healthcare costs. Maternal outcomes include life expectancy and per-person HIV-related healthcare costs. These outcomes are derived from the CEPAC Adult and Pediatric models, through specific simulations of each possible scenario described at the end of the pathways shown in the MTCT model. As an example of infant outcomes from the CEPAC-Pediatric model, an HIV-uninfected infant with an ART-eligible mother who is in postnatal care (and thus on ART) would face monthly risks of HIV infection based on receipt of maternal ART during breastfeeding; if infected postnatally, the infant would face CD4- and age-stratified monthly risks of OIs, ART failure or toxicity, and AIDS-related and AIDS-unrelated death, leading to a LE and lifetime cost projection for the infant. As an example of maternal outcomes from the CEPAC-Adult model, this infant’s mother would face CD4-dependent monthly risks of OIs, ART failure or toxicity, and AIDS-related and AIDS-unrelated death, leading to a LE and lifetime cost projection for herself. These CEPAC model outputs are then used as “payoffs” (outcomes) in the MTCT model, according to conventional methods for evaluation of a decision tree. The average value assigned to any modeled PMTCT strategy in the MTCT model is, in essence, a weighted average of the value of these outcomes at the end of each pathway (weighted by the probabilities of reaching each possible path endpoint). **Abbreviations: ARVs**: antiretroviral drugs; **ART**: three-drug antiretroviral therapy; **ANC**: antenatal care; **AZT**: zidovudine; **sdNVP**: single-dose nevirapine (excluded for this analysis).
(TIFF)

S2 Fig Schematic representations of the CEPAC adult and infant models (adapted with permission from Ciaranello *et al*, *PLoS ONE*, 2011; 6(6)). Schematic representations of the adult and infant CEPAC model structures. Women enter the adult model (S2A Fig.) after delivery; for this analysis, all modeled women enter with chronic HIV infection. They then face monthly risks of clinical events including opportunistic infections, medication toxicities, and death; these risks are stratified by the parameters listed in the figure. Life months accrued between presentation to antenatal care and delivery are added to the CEPAC model projections. Infants enter the pediatric model (S2B Fig.) after delivery; for this analysis, all modeled infants enter either HIV-negative or with an intrauterine/intrapartum HIV infection. Infants HIV-negative upon entering the model can either develop a postpartum HIV infection via breastfeeding or remain HIV-negative. HIV-infected infants face monthly risks of clinical events including opportunistic infections, medication toxicities, and death; these risks are stratified by age and CD4%.
(TIFF)

S1 Table Complete input parameters for a model of mother-to-child transmission in South Africa (includes parameters listed in manuscript Table 1). (DOCX)

S2 Table Intermediate (CEPAC model) results. (DOCX)

S3 Table Sensitivity analyses for the comparison of *POC* and *laboratory* CD4 testing in antenatal care. (DOCX)

S4 Table Cumulative ANC and pediatric costs and pediatric survival over the first five years after delivery (undiscounted; input data for Manuscript Fig. 4). (DOCX)

## References

[1] Joint United Nations Programme on HIV/AIDS (UNAIDS) (2013) UNAIDS report on the global AIDS epidemic. Available: http://www.unaids.org/en/media/unaids/contentassets/documents/epidemiology/2013/gr2013/UNAIDS_Global_Report_2013_en.pdf. Accessed 2014 June 22.

[2] Rollins N, Mahy M, Becquet R, Kuhn L, Creek T, et al (2012) Estimates of peripartum and postnatal mother-to-child transmission probabilities of HIV for use in Spectrum and other population-based models. Sex Transm Infect 88 Suppl 2: i44–51. 10.1136/sextrans-2012-050709 23172345 PMC3512432

[3] World Health Organization (2010) Antiretroviral drugs for treating pregnant women and preventing HIV infection in infants: towards universal access. Geneva, Switzerland: WHO Press. Available: http://whqlibdoc.who.int/publications/2010/9789241599818_eng.pdf. Accessed 2014 July 14.

[4] World Health Organization (2013) Consolidated guidelines on the use of antiretrovirals for the treatment and prevention of HIV infection. Available: http://www.who.int/hiv/pub/guidelines/arv2013/download/en/index.html. Accessed 2014 July 24.

[5] South Africa National Department of Health (2010) Clinical Guidelines: PMTCT (Prevention of Mother-to-Child Transmission). Available: http://www.fidssa.co.za/images/PMTCT_Guidelines.pdf. Accessed 2014 July 12.

[6] South African Department of Health (2013) The South African Antiretroviral Treatment Guidelines. South Africa: South African Department of Health. Available: http://www.doh.gov.za/docs/policy/2013/ART_Treatment_Guidelines_Final_25March2013.pdf. Accessed 2014 July 3.

[7] Ahmed S, Kim MH, Abrams EJ (2013) Risks and benefits of lifelong antiretroviral treatment for pregnant and breastfeeding women: a review of the evidence for the Option B+ approach. Curr Opin HIV AIDS 8: 474–489. 10.1097/COH.0b013e328363a8f2 23925003

[8] Carter RJ, Dugan K, El-Sadr WM, Myer L, Otieno J, et al (2010) CD4+ cell count testing more effective than HIV disease clinical staging in identifying pregnant and postpartum women eligible for antiretroviral therapy in resource-limited settings. J Acquir Immune Defic Syndr 55: 404–410. 10.1097/QAI.0b013e3181e73f4b 20595905

[9] Townsend CL, Cortina-Borja M, Peckham CS, de Ruiter A, Lyall H, et al (2008) Low rates of mother-to-child transmission of HIV following effective pregnancy interventions in the United Kingdom and Ireland, 2000–2006. AIDS 22: 973–981. 10.1097/QAD.0b013e3282f9b67a 18453857

[10] Warszawski J, Tubiana R, Le Chenadec J, Blanche S, Teglas JP, et al (2008) Mother-to-child HIV transmission despite antiretroviral therapy in the ANRS French Perinatal Cohort. AIDS 22: 289–299. 18097232 10.1097/QAD.0b013e3282f3d63c

[11] Chibwesha CJ, Giganti MJ, Putta N, Chintu N, Mulindwa J, et al (2011) Optimal time on HAART for prevention of mother-to-child transmission of HIV. J Acquir Immune Defic Syndr 58: 224–228. 10.1097/QAI.0b013e318229147e 21709566 PMC3605973

[12] Fitzgerald FC, Bekker LG, Kaplan R, Myer L, Lawn SD, et al (2010) Mother-to-child transmission of HIV in a community-based antiretroviral clinic in South Africa. S Afr Med J 100: 827–831. 21414276 10.7196/samj.4045 PMC3954611

[13] Stinson K, Boulle A, Coetzee D, Abrams EJ, Myer L (2010) Initiation of highly active antiretroviral therapy among pregnant women in Cape Town, South Africa. Trop Med Int Health 15: 825–832. 10.1111/j.1365-3156.2010.02538.x 20497405

[14] Myer L, Zulliger R, Black S, Pienaar D, Bekker LG (2012) Pilot programme for the rapid initiation of antiretroviral therapy in pregnancy in Cape Town, South Africa. AIDS Care 24: 986–992. 10.1080/09540121.2012.668173 22519561

[15] Myer L, Daskilewicz K, McIntyre J, Bekker LG (2013) Comparison of point-of-care versus laboratory-based CD4 cell enumeration in HIV-positive pregnant women. J Int AIDS Soc 16: 18649 10.7448/IAS.16.1.18649 24044627 PMC3776301

[16] Jani IV, Sitoe NE, Alfai ER, Chongo PL, Quevedo JI, et al (2011) Effect of point-of-care CD4 cell count tests on retention of patients and rates of antiretroviral therapy initiation in primary health clinics: an observational cohort study. Lancet 378: 1572–1579. 10.1016/S0140-6736(11)61052-0 21951656

[17] Larson B, Schnippel K, Ndibongo B, Xulu T, Brennan A, et al (2012) Rapid point-of-care CD4 testing at mobile HIV testing sites to increase linkage to care: an evaluation of a pilot program in South Africa. J Acquir Immune Defic Syndr 61: e13–17. 22659650 10.1097/QAI.0b013e31825eec60 PMC3458178

[18] Mnyani CN, McIntyre JA, Myer L (2012) The reliability of point-of-care CD4 testing in identifying HIV-infected pregnant women eligible for antiretroviral therapy. J Acquir Immune Defic Syndr 60: 260–264. 10.1097/QAI.0b013e318256b651 22487589

[19] Glencross DK, Coetzee LM, Faal M, Masango M, Stevens WS, et al (2012) Performance evaluation of the Pima point-of-care CD4 analyser using capillary blood sampling in field tests in South Africa. J Int AIDS Soc 15: 3 10.1186/1758-2652-15-3 22284546 PMC3310849

[20] Jani IV, Sitoe NE, Quevedo JI, Lehe JD, Peter TF. Cost comparison of point-of-care and laboratory CD4 testing in resource-limited settings. Abstract #MOAD0101; 2011; Rome, Italy. Available: http://pag.ias2011.org/abstracts.aspx?aid=4201. Accessed 2014 May 1.

[21] Larson B, Schnippel K, Ndibongo B, Long L, Fox MP, et al (2012) How to estimate the cost of point-of-care CD4 testing in program settings: an example using the Alere Pima Analyzer in South Africa. PLoS One 7: e35444 10.1371/journal.pone.0035444 22532854 PMC3331987

[22] Cleary S, Chitha W, Jikwana S, Okorafor OA, Boulle A (2005) Health Systems Trust: South African Health Review. Durban Available: http://www.healthlink.org.za/publications/682. Accessed 2014 July 1.

[23] Myer L (2013) Personal communication regarding treatment data at the Hanover Park Midwife Obstetrics Unit.

[24] Clinton Health Access Initiative (2012) Antiretroviral (ARV) Ceiling Price List. Available: http://d2pd3b5abq75bb.cloudfront.net/2012/07/12/15/03/07/163/CHAI_ARV_Ceiling_Price_List_May_2012.pdf. Accessed 2014 July 18.

[25] Ciaranello AL, Perez F, Engelsmann B, Walensky RP, Mushavi A, et al (2013) Cost-effectiveness of World Health Organization 2010 guidelines for prevention of mother-to-child HIV transmission in Zimbabwe. Clin Infect Dis 56: 430–446. 10.1093/cid/cis858 23204035 PMC3540037

[26] Ciaranello A, Perez F, Keatinge J, Park J, Engelsmann B, et al (2012) What will it take to eliminate pediatric HIV? Reaching “virtual elimination” targets for prevention of mother-to-child HIV transmission (PMTCT) in Zimbabwe. PLoS Med 9: e1001156 10.1371/journal.pmed.1001156 22253579 PMC3254654

[27] Ciaranello AL, Perez F, Maruva M, Chu J, Englesmann B, et al (2011) WHO 2010 guidelines for prevention of mother-to-child HIV transmission in Zimbabwe: Modeling clinical outcomes in infants and mothers. PLoS ONE 6: e20224 10.1371/journal.pone.0020224 21655097 PMC3107213

[28] Ciaranello A, Lockman S, Freedberg KA, Hughes M, Chu J, et al (2011) First-line antiretroviral therapy after single-dose nevirapine exposure in South Africa: a cost-effectiveness analysis of the OCTANE trial. AIDS 25: 479–492. 10.1097/QAD.0b013e3283428cbe 21293199 PMC3068908

[29] Goldie SJ, Yazdanpanah Y, Losina E, Weinstein MC, Anglaret X, et al (2006) Cost-effectiveness of HIV treatment in resource-poor settings—the case of Côte d’Ivoire. N Engl J Med 355: 1141–1153. 16971720 10.1056/NEJMsa060247

[30] Ciaranello AL, Morris BL, Walensky RP, Weinstein MC, Ayaya S, et al (2013) Validation and calibration of a computer simulation model of pediatric HIV infection. PLoS ONE 8: e83389 10.1371/journal.pone.0083389 24349503 PMC3862684

[31] Gold MR, Siegel JE, Russell LB, Weinstein MC, editors (1996) Cost-effectiveness in health and medicine. New York: Oxford University Press.

[32] World Health Organization (2010) WHO-CHOICE: Cost-effectiveness thresholds. Available: www.who.int/choice/costs/CER_thresholds/en/index.html. Accessed 2014 July 13.

[33] International Monetary Fund (IMF) (2014) World Economic Outlook Database. Available: http://www.imf.org/external/ns/cs.aspx?id = 28. Accessed 2014 July 20.

[34] Walensky RP, Wolf LL, Wood R, Fofana MO, Freedberg KA, et al (2009) When to start antiretroviral therapy in resource-limited settings. Ann Intern Med 151: 157–166. 19620143 10.7326/0003-4819-151-3-200908040-00138 PMC3092478

[35] Holmes CB, Wood R, Badri M, Zilber S, Wang B, et al (2006) CD4 decline and incidence of opportunistic infections in Cape Town, South Africa: implications for prophylaxis and treatment. J Acquir Immune Defic Syndr 42: 464–469. 16810113 10.1097/01.qai.0000225729.79610.b7

[36] Ciaranello AL, Lu Z, Ayaya S, Losina E, Musick B, et al (2014) Incidence of WHO Stage 3 and 4 events, tuberculosis, and mortality in untreated, HIV-infected children enrolling in care before 1 year of age: an IeDEA (International Epidemiologic Databases to Evaluate AIDS) East Africa regional analysis. Pediatr Infect Dis J 33: 623–629. 10.1097/INF.0000000000000223 24378935 PMC4024340

[37] South Africa National Department of Health (2010) Antiretroviral Treatment Guidelines. Available: http://www.uj.ac.za/EN/CorporateServices/ioha/Documentation/Documents/ART%20Guideline.pdf. Accessed 2014 July 11.

[38] World Health Organization (2010) Antiretroviral therapy for HIV infection in adults and adolescents—Recommendations for a public health approach. Available: http://www.who.int/hiv/pub/arv/adult2010/en/index.html. Accessed 2014 July 2. 23741771

[39] South African National Department of Health (2010) Guidelines for the management of HIV in children. Available: http://www.sahivsoc.org/upload/documents/Guidelines_for_Management_of_HIV_in_Children_2010.pdf. Accessed 2014 July 20.

[40] Coetzee D, Hildrebrand K, Boulle A, Maartens G, Louis F, et al (2004) Outcomes after two years of providing antiretroviral treatment in Khayelitsha, South Africa. AIDS 18: 887–895. 15060436 10.1097/00002030-200404090-00006

[41] Lockman S, Hughes MD, McIntyre J, Zheng Y, Chipato T, et al (2010) Antiretroviral therapies in women after single-dose nevirapine exposure. N Engl J Med 363: 1499–1509. 10.1056/NEJMoa0906626 20942666 PMC2994321

[42] Stringer JS, McConnell MS, Kiarie J, Bolu O, Anekthananon T, et al (2010) Effectiveness of non-nucleoside reverse-transcriptase inhibitor-based antiretroviral therapy in women previously exposed to a single intrapartum dose of nevirapine: a multi-country, prospective cohort study. PLoS Med 7: e1000233 10.1371/journal.pmed.1000233 20169113 PMC2821896

[43] Violari A, Lindsey JC, Hughes MD, Mujuru HA, Barlow-Mosha L, et al (2012) Nevirapine versus ritonavir-boosted lopinavir for HIV-infected children. N Engl J Med 366: 2380–2389. 10.1056/NEJMoa1113249 22716976 PMC3443859

[44] Palumbo P, Lindsey JC, Hughes MD, Cotton MF, Bobat R, et al (2010) Antiretroviral treatment for children with peripartum nevirapine exposure. N Engl J Med 363: 1510–1520. 10.1056/NEJMoa1000931 20942667 PMC3021781

[45] Black S, Zulliger R, Myer L, Marcus R, Jeneker S, et al (2013) Safety, feasibility and efficacy of a rapid ART initiation in pregnancy pilot programme in Cape Town, South Africa. S Afr Med J 103: 557–562. 10.7196/samj.6565 23885739

[46] Anglaret X, Chene G, Attia A, Toure S, Lafont S, et al (1999) Early chemoprophylaxis with trimethoprim-sulphamethoxazole for HIV-1-infected adults in Abidjan, Côte d’Ivoire: a randomised trial. Cotrimo-CI Study Group. Lancet 353: 1463–1468. 10232311 10.1016/s0140-6736(98)07399-1

[47] Thomas LS (2006) Costing of HIV/AIDS services at a tertiary level hospital in Gauteng Province. Faculty of Health Sciences, University of Witwatersrand, South Africa. Available: http://wiredspace.wits.ac.za/handle/10539/2008. Accessed 2014 May 6.

[48] UNITAID (2014) HIV/AIDS Diagnostics Technology Landscape. Available: http://www.unitaid.org/images/marketdynamics/publications/UNITAID-HIV_Diagnostic_Landscape-4th_edition.pdf. Accessed 2014 October 20.

[49] Palamountain KM, Baker J, Cowan EP, Essajee S, Mazzola LT, et al (2012) Perspectives on introduction and implementation of new point-of-care diagnostic tests. J Infect Dis 205 Suppl 2: S181–190. 10.1093/infdis/jis203 22402038 PMC3334510

[50] Thairu L, Katzenstein D, Israelski D (2011) Operational challenges in delivering CD4 diagnostics in sub-Saharan Africa. AIDS Care 23: 814–821. 10.1080/09540121.2010.541416 21400312

[51] UNAIDS (2012) Report on the global AIDS epidemic Geneva, Switzerland: Joint United Nations Programme on HIV/AIDS. Available: http://www.unaids.org/en/media/unaids/contentassets/documents/epidemiology/2012/gr2012/20121120_UNAIDS_Global_Report_2012_en.pdf. Accessed 2014 July 18.

[52] Goga A, Dinh TH, Jackson D, Lombard C, Crowley S, et al. (2012) Impact of the national prevention of mother-to-child transmission of HIV (PMTCT) program on perinatal mother-to-child transmission of HIV (MTCT) measured at six weeks postpartum, South Africa (SA): results of the first year of implementation of the 2010 P (Abstract no. WEPE173); Washington, D.C. Available: http://www.iasociety.org/Default.aspx?pageId=11&abstractId=200745035. Accessed 2014 June 17.

[53] World Health Organization (2012) Programmatic update: use of antiretroviral drugs for treating pregnant women and preventing HIV infection in infants. Available: http://www.who.int/hiv/pub/mtct/programmatic_update2012/en/. Accessed 2014 July 15.

[54] Schouten EJ, Jahn A, Midiani D, Makombe SD, Mnthambala A, et al (2011) Prevention of mother-to-child transmission of HIV and the health-related Millennium Development Goals: time for a public health approach. Lancet 378: 282–284. 10.1016/S0140-6736(10)62303-3 21763940

[55] Jani IV, Sitoe NE, Chongo PL, Alfai ER, Quevedo JI, et al (2011) Accurate CD4 T-cell enumeration and antiretroviral drug toxicity monitoring in primary healthcare clinics using point-of-care testing. AIDS 25: 807–812. 10.1097/QAD.0b013e328344f424 21378535

[56] Balakrishnan P, Iqbal HS, Shanmugham S, Mohanakrishnan J, Solomon SS, et al (2011) Low-cost assays for monitoring HIV infected individuals in resource-limited settings. Indian J Med Res 134: 823–834. 10.4103/0971-5916.92628 22310816 PMC3284092

[57] Walker AS, Gibb DM (2011) Monitoring of highly active antiretroviral therapy in HIV infection. Curr Opin Infect Dis 24: 27–33. 10.1097/QCO.0b013e3283423e0e 21150591

[58] World Health Organization Department of Maternal, Newborn, Child and Adolescent Health (2014) Does provision of point of care CD4 technology and early knowledge of CD4 levels affect early initiation and retention on anti-retroviral (ART) in HIV positive pregnancy women in the context of Option B+ for PMTCT? Available: http://www.clinicaltrials.gov/ct2/show/NCT02070900. Accessed 2014 July 9.

[59] Kellerman SE, Ahmed S, Feeley-Summerl T, Jay J, Kim M, et al (2013) Beyond prevention of mother-to-child transmission: keeping HIV-exposed and HIV-positive children healthy and alive. AIDS 27 Suppl 2: S225–233. 10.1097/QAD.0000000000000107 24361632 PMC4087192

[60] World Health Organization (2010) New guidance on prevention of mother-to-child transmission of HIV and infant feeding in the context of HIV. Available: http://www.who.int/hiv/pub/mtct/PMTCTfactsheet/en/index.html. Accessed 2014 July 12.

[61] Kesho Bora Study Group (2011) Triple antiretroviral compared with zidovudine and single-dose nevirapine prophylaxis during pregnancy and breastfeeding for prevention of mother-to-child transmission of HIV-1 (Kesho Bora study): a randomised controlled trial. Lancet Infect Dis 1: 159. 10.1016/S1473-3099(10)70288-7 21237718

[62] Kesho Bora Study Group (2012) Maternal HIV-1 disease progression 18–24 months postdelivery according to antiretroviral prophylaxis regimen (triple-antiretroviral prophylaxis during pregnancy and breastfeeding vs zidovudine/single-dose nevirapine prophylaxis): The Kesho Bora randomized controlled trial. Clin Infect Dis 55: 449–460. 10.1093/cid/cis461 22573845 PMC3393708

[63] Connor EM, Sperling RS, Gelber R, Kiselev P, Scott G, et al (1994) Reduction of maternal-infant transmission of human immunodeficiency virus type 1 with zidovudine treatment. Pediatric AIDS Clinical Trials Group Protocol 076 Study Group. N Engl J Med 331: 1173–1180. 7935654 10.1056/NEJM199411033311801

[64] Shapiro RL, Hughes MD, Ogwu A, Kitch D, Lockman S, et al (2010) Antiretroviral regimens in pregnancy and breast-feeding in Botswana. N Engl J Med 362: 2282–2294. 10.1056/NEJMoa0907736 20554983 PMC2999916

[65] National Institutes of Health: IMPAACT Trial Network (2012) The PROMISE Study (Promoting Maternal and Infant Survival Everywhere): Examining Benefits of HAART Continuation in Postpartum Mothers. Available: http://clinicaltrials.gov/show/NCT00955968. Accessed 3 July 2014.

[66] Gopalappa C, Stover J, Shaffer N, Mahy M (2014) The costs and benefits of Option B+ for the prevention of mother-to-child transmission of HIV. AIDS 28 Suppl 1: S5–14. 10.1097/QAD.0000000000000083 24468947

[67] Cohen MS, Chen YQ, McCauley M, Gamble T, Hosseinipour MC, et al (2011) Prevention of HIV-1 infection with early antiretroviral therapy. N Engl J Med 365: 493–505. 10.1056/NEJMoa1105243 21767103 PMC3200068

[68] Walensky RP, Ross EL, Kumarasamy N, Wood R, Noubary F, et al (2013) Cost-effectiveness of HIV treatment as prevention in serodiscordant couples. The New England journal of medicine 369: 1715–1725. 10.1056/NEJMsa1214720 24171517 PMC3913536

[69] Briggs AH, Weinstein MC, Fenwick EA, Karnon J, Sculpher MJ, et al (2012) Model parameter estimation and uncertainty: a report of the ISPOR-SMDM Modeling Good Research Practices Task Force—6. Value Health 15: 835–842. 10.1016/j.jval.2012.04.014 22999133

[70] Stinson K, Myer L (2012) Barriers to initiating antiretroviral therapy during pregnancy: a qualitative study of women attending services in Cape Town, South Africa. African Journal of AIDS Research 11: 65–73. 10.2989/16085906.2012.671263 25870899

[71] Hoffman RM, Black V, Technau K, van der Merwe KJ, Currier J, et al (2010) Effects of highly active antiretroviral therapy duration and regimen on risk for mother-to-child transmission of HIV in Johannesburg, South Africa. J Acquir Immune Defic Syndr 54: 35–41. 10.1097/QAI.0b013e3181cf9979 20216425 PMC2880466

[72] Department of Health Republic of South Africa (2012) Saving mothers 2008–2010: fifth report on the confidential enquiries into maternal deaths in South Africa- short report.

[73] Dabis F, Bequet L, Ekouevi DK, Viho I, Rouet F, et al (2005) Field efficacy of zidovudine, lamivudine and single-dose nevirapine to prevent peripartum HIV transmission. AIDS 19: 309–318. 15718842 PMC2474891

[74] Thior I, Lockman S, Smeaton LM, Shapiro RL, Wester C, et al (2006) Breastfeeding plus infant zidovudine prophylaxis for 6 months vs formula feeding plus infant zidovudine for 1 month to reduce mother-to-child HIV transmission in Botswana: a randomized trial: the Mashi Study. JAMA 296: 794–805. 16905785 10.1001/jama.296.7.794

[75] Kilewo C, Karlsson K, Ngarina M, Massawe A, Lyamuya E, et al (2009) Prevention of mother-to-child transmission of HIV-1 through breastfeeding by treating mothers with triple antiretroviral therapy in Dar es Salaam, Tanzania: the Mitra Plus study. J Acquir Immune Defic Syndr 52: 406–416. 10.1097/QAI.0b013e3181b323ff 19730269

[76] Tonwe-Gold B, Ekouevi DK, Viho I, Amani-Bosse C, Toure S, et al (2007) Antiretroviral treatment and prevention of peripartum and postnatal HIV transmission in West Africa: evaluation of a two-tiered approach. PLoS Med 4: e257 17713983 10.1371/journal.pmed.0040257 PMC1949842

[77] Peltier CA, Ndayisaba GF, Lepage P, van Griensven J, Leroy V, et al (2009) Breastfeeding with maternal antiretroviral therapy or formula feeding to prevent HIV postnatal mother-to-child transmission in Rwanda. AIDS 23: 2415–2423. 10.1097/QAD.0b013e32832ec20d 19730349 PMC3305463

[78] Palombi L, Marazzi MC, Voetberg A, Magid NA (2007) Treatment acceleration program and the experience of the DREAM program in prevention of mother-to-child transmission of HIV. AIDS 21 Suppl 4: S65–71. 17620755 10.1097/01.aids.0000279708.09180.f5

[79] Chasela CS, Hudgens MG, Jamieson DJ, Kayira D, Hosseinipour MC, et al (2010) Maternal or infant antiretroviral drugs to reduce HIV-1 transmission. N Engl J Med 362: 2271–2281. 10.1056/NEJMoa0911486 20554982 PMC3440865

[80] Vyankandondera J, Luchters S, Hassink E. (2003) Reducing risk of HIV-1 transmission from mother to infant through breastfeeding using antiretroviral prophylaxis in infants (SIMBA-study, Abstract N°LB7); Paris, France Available: http://www.iasociety.org/Default.aspx?pageId=11&abstractId=11061. Accessed 2014 May 22.

[81] Thomas TK, Masaba R, Borkowf CB, Ndivo R, Zeh C, et al (2011) Triple-antiretroviral prophylaxis to prevent mother-to-child HIV transmission through breastfeeding—the Kisumu Breastfeeding Study, Kenya: a clinical trial. PLoS Med 8: e1001015 10.1371/journal.pmed.1001015 21468300 PMC3066129

[82] World Health Organization (2010) Antiretroviral therapy for HIV infection in infants and children: Recommendations for a public health approach. 23741772

[83] Vander Plaetse B, Hlatiwayo G, Van Eygen L, Meessen B, Criel B (2005) Costs and revenue of health care in a rural Zimbabwean district. Health Policy Plan 20: 243–251. 15965036 10.1093/heapol/czi028

