# Supplementary material for: Point-of-Care CD4 Testing to Inform Selection of Antiretroviral Medications in South African Antenatal Clinics: A Cost-Effectiveness Analysis
Source: PLoS One. 2015 Mar 10;10(3):e0117751. doi: 10.1371/journal.pone.0117751 (PMC4355621; doi:10.1371/journal.pone.0117751)
Supplement: S1 Appendix — (DOCX) [file pone.0117751.s001.docx]

Point-of-care CD4 Testing to Inform Selection of Antiretroviral Medications in

South African Antenatal Clinics: a Cost-effectiveness Analysis

**Appendix S1**

**Andrea L. Ciaranello, MD, MPH, *et al.***

**INTRODUCTION**

This appendix is included to provide methodologic details to supplement the description of the methods in the manuscript text, as well as additional model output and results. Additional information regarding model structure and data input parameters is also available in the Appendices of prior publications by the authors.^1-3^

**METHODS**

**Pilot evaluation of a point-of-care (POC) CD4 assay in an antenatal clinic**

The Cape Town-based research team utilized a before-and-after study design to evaluate the use of the Pima POC CD4 machine (Alere™) at the Gugulethu Midwife Obstetric Unit (MOU). The MOU serves a population of approximately 300,000 people with an HIV prevalence of 30% in the former township of Gugulethu, a peri-urban setting located 15 km from Cape Town, South Africa.^4^ The Gugulethu MOU is the primary provider of antenatal care (ANC) services in this region and treats approximately 4,600 women annually. Prior to June 1, 2012, laboratory-based CD4 testing was the standard practice. Women booking for ANC underwent rapid HIV testing; those identified as HIV-infected were offered zidovudine (AZT) at this first visit, and specimens were collected and transported to the National Health Laboratory Services (NHLS) in Cape Town for CD4 assessment via flow cytometry. After laboratory processing, CD4 results were made available on an NHLS website within three days, which required nurses to check for specific patient results, and then paper copies of the results were mailed to the MOU approximately three weeks later. After obtaining the results, clinic staff called patients by telephone to return to the clinic to replace AZT with three-drug ART if CD4 was ≤350/µL. A "rapid ART integration" program was also in place at the MOU, which permitted women with CD4 ≤350/µL to undergo intensive counseling and screening and initiate ART in the MOU on their first return visit for receipt of CD4 results, without separate referral to an ART clinic.^5-6^

After a smaller pilot study to assess feasibility,^5^ the *POC* CD4 assay was introduced into routine care in the MOU on June 1, 2012. Women identified as HIV-infected through rapid HIV testing underwent specimen collection, *POC* CD4 assessment, return of CD4 results, and counseling and initiation of ART if CD4 was ≤350/µL during their first visit to the MOU. From January 1-May 31, 2012 (*laboratory*) and June 1- December 31, 2012 (*POC*), data were collected by trained study staff regarding the number of women booking for antenatal care, undergoing HIV testing, and testing HIV-positive; the number and proportion of HIV-infected women undergoing CD4 testing; the proportion of CD4-tested women receiving CD4 results and initiating ART; and the time (days) to receipt of CD4 results and ART initiation. These data were then used as inputs for the various steps of the ANC and prevention of mother-to-child HIV transmission (PMTCT) cascade, which are depicted in greater detail in Figure S1.

**Model structure**

The structures of the Cost-effectiveness of Preventing AIDS Complications (CEPAC) adult, pediatric, and mother-to-child transmission (MTCT) models are described below, and the reader is also referred to several publications providing further detail about model structure and model validation.^2,3,7-8^

The MTCT and CEPAC models are linked to allow a combined analysis in which each woman-infant pair is simulated together from the time of first presentation at ANC through pregnancy and delivery (the MTCT model), and then each woman and infant are simulated separately throughout their lifetimes (the CEPAC models). This linkage was accomplished by first simulating maternal and infant outcomes in the appropriate CEPAC models, then using CEPAC model results (postnatal infection risk, LE, and yearly or lifetime costs) as inputs to the MTCT model (Figure S1).

Antenatal and intrapartum outcomes: the MTCT model (Please see also Figure S1 and the accompanying legend).

The MTCT model is a validated simulation model of a cohort of pregnant women, from the time of conception through delivery. A single pregnancy per woman is simulated. The model is a deterministic model, with a decision-tree structure, coded in TreeAgePro software (Williamstown, MA).^1,2,9-10^ Key modeled events include the steps in the PMTCT "cascade of care:" presentation to ANC; offer and acceptance of HIV testing; receipt of HIV test results; clinical assessment for ART eligibility; CD4 testing and receipt of results; offer of, acceptance of, and adherence to antiretroviral drugs (ARVs) for PMTCT; maternal mortality during pregnancy; HIV testing in labor for women with unknown or negative HIV status; live birth; infant HIV infection by the time of delivery; and linkage to postnatal care and ART for mothers and infants.^11^

Probabilities of HIV transmission by 4-6 weeks of age, reflecting *in utero* and intrapartum HIV transmission, are stratified by maternal HIV stage (CD4 >350/µL or ≤350/µL) and by ARV regimen received for PMTCT. After delivery, model outcomes for infants include vital status and HIV infection/exposure (infected by 4-6 weeks of age or exposed-uninfected); model outcomes for mothers include vital status, CD4 (> or ≤350/µL) and current ART receipt. Model validation analyses and key sensitivity analyses have been reported previously.^1-2^

Postpartum maternal outcomes: the CEPAC adult model (Please also see Figure S2A and the accompanying legend).

The CEPAC International adult model is a first-order, Monte Carlo simulation of HIV infection in adults. HIV-infected women are simulated individually from delivery through death, and HIV disease progression is characterized by monthly transitions between health states. Health states include acute opportunistic infections (OIs), chronic HIV infection, and death. Monthly risks of OIs and HIV-related death are determined by current CD4 count, OI prophylaxis, and history of previous OIs (Table S1, Section Ie). The model records all clinical events and costs accrued monthly over each woman's lifetime; model outcomes include average per-person costs and life expectancy.

Additional structural and technical details of the CEPAC adult model have been reported previously, as have validation analyses comparing model results to published outcomes for postpartum women, as well as men and non-postpartum women.^8,12-14^ The CEPAC website (http://web2.research.partners.org/cepac) also provides details of model structure, including flow charts depicting patient health states and key modeled events, a comprehensive User's Guide to the model, and protocols for deriving model inputs via regular literature reviews.

*Maternal cohort characteristics and disease progression without ART.* At model entry, women are assigned a baseline HIV RNA level (drawn from the distribution observed in the Cape Town AIDS Cohort, Table S1, Section Ia^15^) and a baseline CD4 cell count.^16^ In the absence of effective ART (either before ART initiation or after virologic failure on ART), CD4 counts are modeled to decline at a rate determined by current RNA level (Table S1, Section Ie). Current CD4 count, opportunistic infection (OI) prophylaxis, and history or absence of previous OIs determine the monthly risk of OIs and HIV-related death. HIV-related risks of death in the CEPAC model include mortality risks associated with acute opportunistic infection and chronic HIV-infection. Additional risks of death are derived from age- and gender-specific South African mortality rates.^17^

*Opportunistic infection prophylaxis.* In addition to antiretroviral therapy, all simulated patients receive trimethoprim-sulfamethoxazole as prophylaxis against *Pneumocystis jiroveci* pneumonia and other bacterial infections, and continue this therapy lifelong.^18^ The impact of trimethoprim-sulfamethoxazole prophylaxis on risks of clinical events, including medication toxicities, are described in Table S1, Section Ie.

*Antiretroviral therapy.* After delivery, women not yet on ART are modeled to initiate ART when CD4 falls to ≤350/µL or when a WHO Stage 3 or 4 event occurs. With effective ART, modeled HIV RNA suppression leads to CD4 count increases, reducing the monthly risks for OIs and death. Virologic failure on ART may occur either “early” (≤6 months) or “late” (>6 months) after ART initiation. For patients with early or late virologic failure, CD4 counts decline after a user-specified delay (usually 12 months), accompanied by increased risks of OIs and death. Patients who remain on ART despite virologic failure experience lower risks of OIs and death than do patients who discontinue ART, reflecting the CD4-independent benefit of ART.^19^

In this analysis, based on 2010 South African guidelines during the study period (2010-2013), women in HIV-related care were assumed to undergo outpatient clinical evaluation every three months and CD4 monitoring biannually (pre-ART initiation) and annually (post-ART initiation). HIV RNA monitoring was assumed not to be available pre-ART initiation, but was conducted every 6 months for the first year on ART and every 12 months thereafter.^20^ ART regimens reflected 2010 South Africa guidelines and common current practice in South Africa, depending on timing of ART initiation (Table S1, Section Ie): first-line ART regimens included nevirapine (NVP) or efavirenz (EFV) with tenofovir and emtricitabine (TDF/FTC).^20^ Following WHO recommendations, simulated patients were switched from the first to a second antiretroviral regimen after observed clinical, immunologic, or virologic failure, defined as at least one severe OI, a ≥50% decrease from the peak on-ART CD4 count, an absolute CD4 count <100 cells/µL, or HIV RNA ≥3,000 copies/mL.^21^ Opportunistic infections diagnosed during the first six months of therapy were not considered as criteria for switching or discontinuing therapy, to allow adequate time for the immunologic benefit of ART to develop. If a second ART regimen was needed, it was comprised of zidovudine, lamivudine, and lopinavir/ritonavir (LPV/r).^20^ After failure of 2^nd^-line ART, defined as three severe opportunistic infections or a ≥90% decrease from peak on-ART CD4 count, this 2^nd^-line regimen was continued unless severe toxicity occurred.^19,21^ To be conservative with regard to the benefit of POC CD4 testing, the maternal health impact of replacing AZT with ART during pregnancy was excluded from this analysis.^22^

Postnatal outcomes: the CEPAC-Pediatric model (Please also see Figure S2B and the accompanying legend).

The CEPAC-Pediatric model is a detailed, individual patient-level microsimulation of HIV infection in children, calibrated to southern African settings.^7^ At birth, infants enter the CEPAC Pediatric model, which uses a random number generator to assign user-specified distributions of CD4% and HIV RNA level. The model uses CD4% for children <5 years of age, and CD4 for children ≥5 years of age. Disease progression follows monthly transitions among numerous health states, including chronic HIV infection, acute illness, and death, and these transitions are dependent upon current CD4% and age. Each month, the simulated infants also face risks of acute clinical events, such as opportunistic infections. In this analysis, we examined three mutually exclusive categories of clinical events: WHO Stage 3, WHO Stage 4, and TB.^23^

*Pediatric cohort characteristics and disease progression without ART.* At birth, infants are assigned a baseline HIV RNA level and a baseline CD4% (Table S1, Section Id). In the absence of effective ART, CD4% is modeled to decline at a rate determined by current age and CD4% until age 5. For this analysis, among infants infected *in utero* or intrapartum, monthly CD4% decline was 4.0% for ages 0-2 months, followed by a monthly decline of 0.5% until age 5 for infants infected intrauterine (IU).^7^ For infants infected postpartum (PP), there was a monthly CD4% decline of 0.5% from ages 0-5 years. At age 5, children transition from a CD4% to an absolute CD4 count, at which point their CD4 decline follows that of the adult CEPAC model. Current CD4%, opportunistic infection (OI) prophylaxis, and history or absence of previous OIs determine the monthly risk of OIs and HIV-related death. HIV-related risks of death in the CEPAC model include mortality risks associated with acute opportunistic infection and chronic HIV infection. Additional risks of death are derived from age- and gender-specific South African mortality rates.^24^ In the CEPAC-Pediatric model, the additional risks of death are specified separately for HIV-unexposed infants, HIV-exposed, uninfected infants, and HIV-infected infants.^24^

*Antiretroviral therapy.* For this analysis, following WHO and South African pediatric ART guidelines, all HIV-infected infants are assumed to undergo rapid HIV diagnosis and to start ART immediately.^25,26^ For each ART regimen, the probability of initial suppression, late failure, and costs are specified separately for children of various ages (Table S1, Section Id). Here, we model two lines of available pediatric ART. First-line ART is abacavir/ lamivudine/lopinavir/ritonavir (ABC/3TC/LPV/r), with those who fail receiving nevirapine (NVP, age <3 years) or efavirenz (EFV, age ≥3 years) with zidovudine/lamivudine (AZT/3TC) for second-line ART.^20^ Simulated patients are switched from the first to a second antiretroviral regimen after observed clinical, immunologic, or virologic failure, defined as at least one severe OI, a CD4% <10% , or HIV RNA ≥5,000 copies/mL after ≥6 months on ART. Children who are observed as failing their second-line ART regimen continue on their second-line regimen lifelong, yet have lower risks of OIs and death than do patients who discontinue ART, reflecting the CD4-independent benefit of ART.^19^

**Loss to follow-up in the linked models**

In the base-case analyses, we assumed guideline-concordant care, including 100% retention in care throughout pregnancy, the postpartum period, and lifelong. In sensitivity analyses, we examined the impact of loss to follow-up for mothers and infants, to better reflect current programmatic experiences. Loss to follow-up in the linked models can occur between ANC booking and delivery, between delivery and six weeks postpartum,^4,27-35^ or after linkage to postnatal HIV care.^27-28,36-41^

**Sensitivity analyses**

Because of their varied structures, each of the models used in this analysis permits different techniques for the evaluation of uncertainty (for example, Markov models and decision trees permit probabilistic sensitivity analyses, while Monte Carlo models do not readily facilitate this methodology).^42^ In order to apply consistent methods for conducting sensitivity analyses among all of the linked models, and to increase transparency and comprehensibility for the reader, we followed the guidance of the US Panel on Cost-effectiveness in Health and Medicine and the International Society for Pharmacoeconomics and Outcomes Research^43-44^ and conducted extensive univariate and multivariate sensitivity analyses on model parameters shown to be influential in prior work and considered to be potentially influential for this analysis.

**Clinical model input data**

*MTCT risks (Manuscript Table 1 and Appendix Table S1, Section Ic).* MTCT risks for each modeled regimen are derived from key PMTCT studies in breastfeeding populations in Africa.^22,30,45-55^ Intrauterine/ intrapartum (IU/IP) MTCT risks were derived from infant HIV PCR results at 4-6 weeks of age, and postnatal MTCT rates were derived from infant PCR results at the latest time point reflecting exposure to both breastmilk and maternal/infant ARVs.^22,30,45-54,56^ MTCT risks during the intrauterine and intrapartum period (by 4-6 weeks of age) and postpartum period (6 weeks-18 months) are stratified by maternal HIV stage and by PMTCT regimen received. The base-case analysis uses the average of published MTCT risks for each regimen. We also examined scenarios in which we used the lowest and highest published risks for each combination of regimen and maternal CD4; these ranges encompass a variety of clinical scenarios, including earlier or later presentation to ANC. In the base case, postpartum transmission risks are based on exclusive breastfeeding (EBF) during the first six months of life.^57^

*Maternal and pediatric HIV disease progression.* Detailed characteristics of maternal and pediatric HIV disease progression can be found in Table S1.

**Economic model input data**

*Antenatal care costs (Manuscript Table 1 and Appendix Table S1, Section IIa and IIb).* We derived routine ANC costs per pregnant woman by dividing the total system costs of the Gugulethu MOU by the number of women receiving ANC in 2012.^58^ In addition to routine care costs, additional costs associated with ANC include weekly ARV medication costs^59^ and non-CD4 laboratory costs (finger-stick hemoglobin, rapid plasminogen reagent (RPR), and rapid HIV test for all patients; creatinine for patients initiating ART).^60^

*Maternal HIV-related healthcare costs*. Maternal HIV-related healthcare costs include four key components: routine care, care for acute clinical events, medications, and laboratory evaluations. We calculated healthcare costs for acute clinical events by estimating the resource utilization (inpatient and outpatient days) for each event, then multiplying by estimated costs for those healthcare visits in South Africa.^60-61^

*Pediatric healthcare costs*. Pediatric HIV-related healthcare costs include four key components: routine care, care for acute clinical events, medications, and laboratory evaluations. For all ages, routine care costs are stratified by CD4% and calculated by estimating resource utilization, then multiplying by estimated costs for these events.^61^ Costs for acute clinical events are stratified by age, with children under five accruing costs for each acute TB, WHO Stage 3, and WHO Stage 4 event.^62^ Children ages 5-13 are assumed to accrue costs for the same acute events as adults.^61^ Through age 13, HIV-infected children receive quarterly CD4 tests before ART initiation. Following ART initiation, HIV-infected children receive CD4 and HIV RNA tests at the same frequency and costs as HIV-infected adults.^60^ After the age of thirteen, children transition to the adult CEPAC model and are assumed to accrue adult care costs.

**RESULTS**

**Intermediate outcomes: CEPAC Model Results (Table S2)**

Selected results of the CEPAC-Adult and CEPAC-Pediatric models are shown in Table S2. Discounted and undiscounted life expectancies and per-person HIV-related healthcare costs were generated from the CEPAC-Adult and CEPAC-Pediatric models for key categories of mothers and infants, and used as inputs to the MTCT model. For women surviving pregnancy, three life-months were added to maternal life expectancy to account for the time between presentation to antenatal care and delivery.

**Sensitivity analyses (Manuscript Table 3 and Appendix Table S3)**

Results of the cost-effectiveness analyses for the base case and key sensitivity analyses are shown in Table S3. The first four sets of parameters in Table S3 are included to provide full numerical results for the sensitivity analyses presented in the manuscript text and manuscript Figures 2 and 3(laboratory CD4 uptake, POC assay cost, POC CD4 uptake, and POC assay sensitivity and specificity). Additional sensitivity analyses include the following (Table S3):

*Maternal loss to follow-up.* Sensitivity analyses were performed using maternal loss to follow-up rates of 0.1187%/month, 0.8268%/month, and 0.8268%/month pre-ART initiation and 0.1187%/month post-ART initiation.^63^ At all of these maternal loss to follow-up rates, *POC* remained cost-saving compared to *laboratory* testing.

*Pediatric loss to follow-up.* Sensitivity analyses were performed using pediatric loss to follow-up rates of 0.4%/month and 0.8%/month.^10^ At both of these pediatric loss to follow-up rates *POC* remained cost-saving compared to *laboratory* testing.

*Breastfeeding duration*. A sensitivity analysis was performed using a breastfeeding duration of 12 months. At this breastfeeding duration, *POC* remained cost-saving compared to *laboratory* testing.

*Healthcare costs*. Sensitivity analyses were performed both doubling and halving all maternal and pediatric healthcare costs. For both doubled and halved healthcare costs, *POC* remained cost-saving compared to *laboratory* testing.

*Medication costs*. Sensitivity analyses were performed both doubling and halving all maternal and pediatric medication costs. For both doubled and halved medication costs, *POC* remained cost-saving compared to *laboratory* testing.

*MTCT risks*. Sensitivity analyses were performed both doubling and halving all MTCT risks. For both doubled and halved MTCT risks, *POC* remained cost-saving compared to *laboratory* testing.

*Discount rate*. Sensitivity analyses were performed using a discount rate of 20% to reflect potentially high borrowing costs. At this discount rate, *POC* testing was equally effective and less expensive compared to *laboratory* testing.

**Budget impact analysis (Table S4)**

The cumulative costs of POC CD4 testing, laboratory testing, and “low laboratory access” testing over the first five years after delivery can be seen in Table S4.

**REFERENCES:**

1. Ciaranello A, Perez F, Keatinge J, Park J, Engelsmann B, et al. (2012) What will it take to eliminate pediatric HIV? Reaching “virtual elimination” targets for prevention of mother-to-child HIV transmission (PMTCT) in Zimbabwe. PLoS Med 9: e1001156.

2. Ciaranello AL, Perez F, Maruva M, Chu J, Englesmann B, et al. (2011) WHO 2010 guidelines for prevention of mother-to-child HIV transmission in Zimbabwe: Modeling clinical outcomes in infants and mothers. PLoS ONE 6: e20224.

3. Ciaranello AL, Perez F, Engelsmann B, Walensky RP, Mushavi A, et al. (2013) Cost-effectiveness of World Health Organization 2010 guidelines for prevention of mother-to-child HIV transmission in Zimbabwe. Clin Infect Dis 56: 430-446.

4. Stinson K, Boulle A, Coetzee D, Abrams EJ, Myer L (2010) Initiation of highly active antiretroviral therapy among pregnant women in Cape Town, South Africa. Trop Med Int Health 15: 825-832.

5. Myer L, Zulliger R, Black S, Pienaar D, Bekker LG (2012) Pilot programme for the rapid initiation of antiretroviral therapy in pregnancy in Cape Town, South Africa. AIDS Care 24: 986-992.

6. Black S, Zulliger R, Myer L, Marcus R, Jeneker S, et al. (2013) Safety, feasibility and efficacy of a rapid ART initiation in pregnancy pilot programme in Cape Town, South Africa. S Afr Med J 103: 557-562.

7. Ciaranello AL, Morris BL, Walensky RP, Weinstein MC, Ayaya S, et al. (2013) Validation and calibration of a computer simulation model of pediatric HIV infection. PLoS ONE 8: e83389.

8. Walensky RP, Wood R, Ciaranello AL, Paltiel AD, Lorenzana SB, et al. (2010) Scaling up the 2010 World Health Organization HIV Treatment Guidelines in resource-limited settings: a model-based analysis. PLoS Med 7: e1000382.

9. Ciaranello AL, Seage GR, 3rd, Freedberg KA, Weinstein MC, Lockman S, et al. (2008) Antiretroviral drugs for preventing mother-to-child transmission of HIV in sub-Saharan Africa: balancing efficacy and infant toxicity. AIDS 22: 2359-2369.

10. Ciaranello A, Chang Y, Margulis A, Bernstein A, Bassett IV, et al. (2009) Effectiveness of pediatric ART in resource-limited settings: a systematic review and meta-analysis. Clin Infect Dis 49: 1915-1927.

11. Barker PM, Mphatswe W, Rollins N (2010) Antiretroviral drugs in the cupboard are not enough: the impact of health systems' performance on mother-to-child transmission of HIV. J Acquir Immune Defic Syndr 56: e45-48.

12. Goldie SJ, Yazdanpanah Y, Losina E, Weinstein MC, Anglaret X, et al. (2006) Cost-effectiveness of HIV treatment in resource-poor settings--the case of Côte d'Ivoire. N Engl J Med 355: 1141-1153.

13. Ciaranello A, Lockman S, Freedberg KA, Hughes M, Chu J, et al. (2011) First-line antiretroviral therapy after single-dose nevirapine exposure in South Africa: a cost-effectiveness analysis of the OCTANE trial. AIDS 25: 479-492.

14. Rydzak CE, Cotich KL, Sax PE, Hsu HE, Wang B, et al. (2010) Assessing the performance of a computer-based policy model of HIV and AIDS. PLoS ONE 5: e12647.

15. Holmes CB, Zheng H, Martinson NA, Freedberg KA, Walensky RP (2006) Optimizing treatment for HIV-infected South African women exposed to single-dose nevirapine: balancing efficacy and cost. Clin Infect Dis 42: 1772-1780.

16. Iliff PJ, Piwoz EG, Tavengwa NV, Zunguza CD, Marinda ET, et al. (2005) Early exclusive breastfeeding reduces the risk of postnatal HIV-1 transmission and increases HIV-free survival. AIDS 19: 699-708.

17. UNAIDS/WHO Working Group on Global HIV/AIDS and STI Surveillance (2009) Epidemiological fact sheet on HIV and AIDS Zimbabwe

18. World Health Organization (2006) World Health Organization: Guidelines for cotrimoxazole prophylaxis for HIV-related infections in children, adolescents, and adults in resource-limited settings.

19. Losina E, Yazdanpanah Y, Deuffic-Burban S, Wang B, Wolf LL, et al. (2007) The independent effect of highly active antiretroviral therapy on severe opportunistic disease incidence and mortality in HIV-infected adults in Côte d'Ivoire. Antivir Ther 12: 543-551.

20. South Africa National Department of Health (2010) Antiretroviral Treatment Guidelines. Available: http://www.uj.ac.za/EN/CorporateServices/ioha/Documentation/Documents/ART%20Guideline.pdf. Accessed 11 July 2014.

21. World Health Organization (2010) Antiretroviral therapy for HIV infection in adults and adolescents - Recommendations for a public health approach. Available: http://www.who.int/hiv/pub/arv/adult2010/en/index.html. Accessed 2 July 2014.

22. Connor EM, Sperling RS, Gelber R, Kiselev P, Scott G, et al. (1994) Reduction of maternal-infant transmission of human immunodeficiency virus type 1 with zidovudine treatment. Pediatric AIDS Clinical Trials Group Protocol 076 Study Group. N Engl J Med 331: 1173-1180.

23. Ciaranello AL, Lu Z, Ayaya S, Losina E, Musick B, et al. (2014) Incidence of WHO Stage 3 and 4 events, tuberculosis, and mortality in untreated, HIV-infected children enrolling in care before 1 year of age: an IeDEA (International Epidemiologic Databases to Evaluate AIDS) East Africa regional analysis. Pediatr Infect Dis J 33: 623-629.

24. United Nations (2009) World Population Prospects: The 2008 Revision. In: Department of Economic and Social Affairs PD, editor. New York.

25. World Health Organization (2010) Antiretroviral therapy for HIV infection in infants and children: Recommendations for a public health approach.

26. South African National Department of Health (2010) Guidelines for the management of HIV in children. Available: http://www.sahivsoc.org/upload/documents/Guidelines_for_Management_of_HIV_in_Children_2010.pdf. Accessed 20 July 2014.

27. Ahoua L, Ayikoru H, Gnauck K, Odaru G, Odar E, et al. (2010) Evaluation of a 5-year programme to prevent mother-to-child transmission of HIV infection in Northern Uganda. J Trop Pediatr 56: 43-52.

28. Manzi M, Zachariah R, Teck R, Buhendwa L, Kazima J, et al. (2005) High acceptability of voluntary counselling and HIV-testing but unacceptable loss to follow up in a prevention of mother-to-child HIV transmission programme in rural Malawi: scaling-up requires a different way of acting. Trop Med Int Health 10: 1242-1250.

29. Kaplan R, Orrell C, Zwane E, Bekker LG, Wood R (2008) Loss to follow-up and mortality among pregnant women referred to a community clinic for antiretroviral treatment. AIDS 22: 1679-1681.

30. Peltier CA, Ndayisaba GF, Lepage P, van Griensven J, Leroy V, et al. (2009) Breastfeeding with maternal antiretroviral therapy or formula feeding to prevent HIV postnatal mother-to-child transmission in Rwanda. AIDS 23: 2415-2423.

31. Kumwenda J, Mataya R, Kumwenda N, Kafulafula G, Li Q, et al. Coverage of highly active antiretroviral therapy (HAART) among postpartum women in Malawi (Abstract WEPDD106); 2009; Cape Town, South Africa. Available: http://www.ias2009.org/pag/Abstracts.aspx?AID=1938. Accessed 27 June 2014.

32. Ramdhial R, Ramkissoon A. WEPDD104: PMTCT: integration of HAART into public sector antenatal care services in a high prevalence HIV setting in South Africa; 2009; Cape Town, South Africa. Available: http://www.iasociety.org/Default.aspx?pageId=12&abstractId=200722054. Accessed 20 July 2014.

33. Srikewal J, Moodley D, Msweli L. The impact of an integrated health system on the delivery of PMTCT services in Kwazulu Natal 2009; Cape Town, South Africa. Available: http://www.ias2009.org/pag/Abstracts.aspx?AID=3432. Accessed 27 July 2014.

34. Stringer EM, Ekouevi DK, Coetzee D, Tih PM, Creek TL, et al. (2010) Coverage of nevirapine-based services to prevent mother-to-child HIV transmission in 4 African countries. JAMA 304: 293-302.

35. Chi BH, Chintu N, Lee A, Stringer EM, Sinkala M, et al. (2007) Expanded services for the prevention of mother-to-child HIV transmission: field acceptability of a pilot program in Lusaka, Zambia. J Acquir Immune Defic Syndr 45: 125-127.

36. Brinkhof MW, Dabis F, Myer L, Bangsberg DR, Boulle A, et al. (2008) Early loss of HIV-infected patients on potent antiretroviral therapy programmes in lower-income countries. Bull World Health Organ 86: 559-567.

37. Geng EH, Bangsberg DR, Musinguzi N, Emenyonu N, Bwana MB, et al. (2010) Understanding reasons for and outcomes of patients lost to follow-up in antiretroviral therapy programs in Africa through a sampling-based approach. J Acquir Immune Defic Syndr 53: 405-411.

38. Amuron B, Namara G, Birungi J, Nabiryo C, Levin J, et al. (2009) Mortality and loss-to-follow-up during the pre-treatment period in an antiretroviral therapy programme under normal health service conditions in Uganda. BMC Public Health 9: 290.

39. Myer L, Carter RJ, Katyal M, Toro P, El-Sadr WM, et al. Impact of antiretroviral therapy on incidence of pregnancy among HIV-infected women in Sub-Saharan Africa: a cohort study. PLoS Med 7: e1000229.

40. Rosen S, Fox MP, Gill CJ (2007) Patient retention in antiretroviral therapy programs in sub-Saharan Africa: a systematic review. PLoS Med 4: e298.

41. Toro PL, Katyal M, Carter RJ, Myer L, El-Sadr WM, et al. (2010) Initiation of antiretroviral therapy among pregnant women in resource-limited countries: CD4+ cell count response and program retention. AIDS 24: 515-524.

42. Hunink MGM, Glasziou PP, Siegel JE, Weeks JC, Pliskin JS, et al. (2003) Decision making in health and medicine: integrating evidence and values. Cambridge: Cambridge University Press.

43. Weinstein MC, Siegel JE, Gold MR, Kamlet MS, Russell LB (1996) Recommendations of the Panel on Cost-effectiveness in Health and Medicine. JAMA 276: 1253-1258.

44. Briggs AH, Weinstein MC, Fenwick EA, Karnon J, Sculpher MJ, et al. (2012) Model parameter estimation and uncertainty: a report of the ISPOR-SMDM Modeling Good Research Practices Task Force--6. Value Health 15: 835-842.

45. Dabis F, Bequet L, Ekouevi DK, Viho I, Rouet F, et al. (2005) Field efficacy of zidovudine, lamivudine and single-dose nevirapine to prevent peripartum HIV transmission. AIDS 19: 309-318.

46. Thior I, Lockman S, Smeaton LM, Shapiro RL, Wester C, et al. (2006) Breastfeeding plus infant zidovudine prophylaxis for 6 months vs formula feeding plus infant zidovudine for 1 month to reduce mother-to-child HIV transmission in Botswana: a randomized trial: the Mashi Study. JAMA 296: 794-805.

47. Kilewo C, Karlsson K, Ngarina M, Massawe A, Lyamuya E, et al. (2009) Prevention of mother-to-child transmission of HIV-1 through breastfeeding by treating mothers with triple antiretroviral therapy in Dar es Salaam, Tanzania: the Mitra Plus study. J Acquir Immune Defic Syndr 52: 406-416.

48. Shapiro RL, Hughes MD, Ogwu A, Kitch D, Lockman S, et al. (2010) Antiretroviral regimens in pregnancy and breast-feeding in Botswana. N Engl J Med 362: 2282-2294.

49. Kesho Bora Study Group (2011) Triple antiretroviral compared with zidovudine and single-dose nevirapine prophylaxis during pregnancy and breastfeeding for prevention of mother-to-child transmission of HIV-1 (Kesho Bora study): a randomised controlled trial. Lancet Infect Dis 1: 159.

50. Tonwe-Gold B, Ekouevi DK, Viho I, Amani-Bosse C, Toure S, et al. (2007) Antiretroviral treatment and prevention of peripartum and postnatal HIV transmission in West Africa: evaluation of a two-tiered approach. PLoS Med 4: e257.

51. Kesho Bora Study Group (2012) Maternal HIV-1 disease progression 18-24 months postdelivery according to antiretroviral prophylaxis regimen (triple-antiretroviral prophylaxis during pregnancy and breastfeeding vs zidovudine/single-dose nevirapine prophylaxis): The Kesho Bora randomized controlled trial. Clin Infect Dis 55: 449-460.

52. Palombi L, Marazzi MC, Voetberg A, Magid NA (2007) Treatment acceleration program and the experience of the DREAM program in prevention of mother-to-child transmission of HIV. AIDS 21 Suppl 4: S65-71.

53. Chasela CS, Hudgens MG, Jamieson DJ, Kayira D, Hosseinipour MC, et al. (2010) Maternal or infant antiretroviral drugs to reduce HIV-1 transmission. N Engl J Med 362: 2271-2281.

54. Vyankandondera J, Luchters S, Hassink E. Reducing risk of HIV-1 transmission from mother to infant through breastfeeding using antiretroviral prophylaxis in infants (SIMBA-study, Abstract N°LB7); 2003; Paris, France. Available: http://www.iasociety.org/Default.aspx?pageId=11&abstractId=11061. Accessed 22 May 2014.

55. Thomas TK, Masaba R, Borkowf CB, Ndivo R, Zeh C, et al. (2011) Triple-antiretroviral prophylaxis to prevent mother-to-child HIV transmission through breastfeeding--the Kisumu Breastfeeding Study, Kenya: a clinical trial. PLoS Med 8: e1001015.

56. Thomas T MR, Ndivo R , Zeh C, Borkowf C, Thigpen M, De Cock K, Amornkul P, Greenberg A , Fowler M, and Kisumu Breastfeeding Study Team. 45aLB: Prevention of mother-to-child transmission of HIV-1 among breastfeeding mothers using HAART: The Kisumu Breastfeeding Study, Kisumu, Kenya, 2003–2007; 2008; Boston. Available: http://www.retroconference.org/2008/Abstracts/33397.htm. Accessed 24 July 2014.

57. Coutsoudis A, Pillay K, Spooner E, Kuhn L, Coovadia HM (1999) Influence of infant-feeding patterns on early mother-to-child transmission of HIV-1 in Durban, South Africa: a prospective cohort study. South African Vitamin A Study Group. Lancet 354: 471-476.

58. Myer L (2013) Personal communication regarding treatment data at the Hanover Park Midwife Obstetrics Unit.

59. Clinton Health Access Initiative (2012) Antiretroviral (ARV) Ceiling Price List. Available: http://d2pd3b5abq75bb.cloudfront.net/2012/07/12/15/03/07/163/CHAI_ARV_Ceiling_Price_List_May_2012.pdf. Accessed 18 July 2014.

60. Cleary S, Chitha W, Jikwana S, Okorafor OA, Boulle A (2005) Health Systems Trust: South African Health Review. Durban. Available: http://www.healthlink.org.za/publications/682. Accessed 1 July 2014.

61. Anglaret X, Chene G, Attia A, Toure S, Lafont S, et al. (1999) Early chemoprophylaxis with trimethoprim-sulphamethoxazole for HIV-1-infected adults in Abidjan, Côte d'Ivoire: a randomised trial. Cotrimo-CI Study Group. Lancet 353: 1463-1468.

62. Thomas LS (2006) Costing of HIV/AIDS services at a tertiary level hospital in Gauteng Province. Faculty of Health Sciences, University of Witwatersrand, South Africa. Available: http://wiredspace.wits.ac.za/handle/10539/2008. Accessed 6 May 2014.

63. Fox MP, Rosen S (2010) Patient retention in antiretroviral therapy programs up to three years on treatment in sub-Saharan Africa, 2007-2009: systematic review. Trop Med Int Health 15 Suppl 1: 1-15.

64. Department of Health Republic of South Africa (2012) Saving mothers 2008-2010: fifth report on the confidential enquiries into maternal deaths in South Africa- short report.

65. Lawn SD, Myer L, Orrell C, Bekker LG, Wood R (2005) Early mortality among adults accessing a community-based antiretroviral service in South Africa: implications for programme design. AIDS 19: 2141-2148.

66. UNAIDS (2012) Report on the global AIDS epidemic Geneva, Switzerland: Joint United Nations Programme on HIV/AIDS. Available: http://www.unaids.org/en/media/unaids/contentassets/documents/epidemiology/2012/gr2012/20121120_UNAIDS_Global_Report_2012_en.pdf. Accessed 18 July 2014.

67. Mnyani CN, McIntyre JA, Myer L (2012) The reliability of point-of-care CD4 testing in identifying HIV-infected pregnant women eligible for antiretroviral therapy. J Acquir Immune Defic Syndr 60: 260-264.

68. Palumbo P, Lindsey JC, Hughes MD, Cotton MF, Bobat R, et al. (2010) Antiretroviral treatment for children with peripartum nevirapine exposure. N Engl J Med 363: 1510-1520.

69. Violari A, Lindsey JC, Hughes MD, Mujuru HA, Barlow-Mosha L, et al. (2012) Nevirapine versus ritonavir-boosted lopinavir for HIV-infected children. N Engl J Med 366: 2380-2389.

70. Mellors JW, Munoz A, Giorgi JV, Margolick JB, Tassoni CJ, et al. (1997) Plasma viral load and CD4+ lymphocytes as prognostic markers of HIV-1 infection. Ann Intern Med 126: 946-954.

71. Holmes CB, Wood R, Badri M, Zilber S, Wang B, et al. (2006) CD4 decline and incidence of opportunistic infections in Cape Town, South Africa: implications for prophylaxis and treatment. J Acquir Immune Defic Syndr 42: 464-469.

72. Yazdanpanah Y, Losina E, Anglaret X, Goldie SJ, Walensky RP, et al. (2005) Clinical impact and cost-effectiveness of co-trimoxazole prophylaxis in patients with HIV/AIDS in Côte d'Ivoire: a trial-based analysis. AIDS 19: 1299-1308.

73. Lockman S, Hughes MD, McIntyre J, Zheng Y, Chipato T, et al. (2010) Antiretroviral therapies in women after single-dose nevirapine exposure. N Engl J Med 363: 1499-1509.

74. Tuboi SH, Brinkhof MW, Egger M, Stone RA, Braitstein P, et al. (2007) Discordant responses to potent antiretroviral treatment in previously naive HIV-1-infected adults initiating treatment in resource-constrained countries: the antiretroviral therapy in low-income countries (ART-LINC) collaboration. J Acquir Immune Defic Syndr 45: 52-59.

75. Gallant JE, DeJesus E, Arribas JR, Pozniak AL, Gazzard B, et al. (2006) Tenofovir DF, emtricitabine, and efavirenz vs. zidovudine, lamivudine, and efavirenz for HIV. N Engl J Med 354: 251-260.

76. Johnson M, Grinsztejn B, Rodriguez C (2005) Atazanavir plus ritonavir or saquinavir, and lopinavir/ritonavir in patients experiencing multiple virological failures. AIDS 19: 685-694.

77. Murphy RA, Sunpath H, Lu Z, Chelin N, Losina E, et al. (2010) Outcomes after virologic failure of first-line ART in South Africa. AIDS 24: 1007-1012.

78. Larson B, Schnippel K, Ndibongo B, Long L, Fox MP, et al. (2012) How to estimate the cost of point-of-care CD4 testing in program settings: an example using the Alere Pima Analyzer in South Africa. PLoS One 7: e35444.

79. Bassett IV, Giddy J, Nkera J, Wang B, Losina E, et al. (2007) Routine voluntary HIV testing in Durban, South Africa: the experience from an outpatient department. J Acquir Immune Defic Syndr 46: 181-186.

80. Walensky RP, Wood R, Fofana MO, Martinson NA, Losina E, et al. (2011) The clinical impact and cost-effectiveness of routine, voluntary HIV screening in South Africa. J Acquir Immune Defic Syndr 56: 26-35.

81. Vander Plaetse B, Hlatiwayo G, Van Eygen L, Meessen B, Criel B (2005) Costs and revenue of health care in a rural Zimbabwean district. Health Policy Plan 20: 243-251.

82. World Health Organization (2010) Antiretroviral drugs for treating pregnant women and preventing HIV infection in infants: towards universal access. Geneva, Switzerland: WHO Press. Available: http://whqlibdoc.who.int/publications/2010/9789241599818_eng.pdf. Accessed 14 July 2014.
