# Supplementary figures and images for: Point-of-Care CD4 Testing to Inform Selection of Antiretroviral Medications in South African Antenatal Clinics: A Cost-Effectiveness Analysis
Source: PLoS One. 2015 Mar 10;10(3):e0117751. doi: 10.1371/journal.pone.0117751 (PMC4355621; doi:10.1371/journal.pone.0117751)

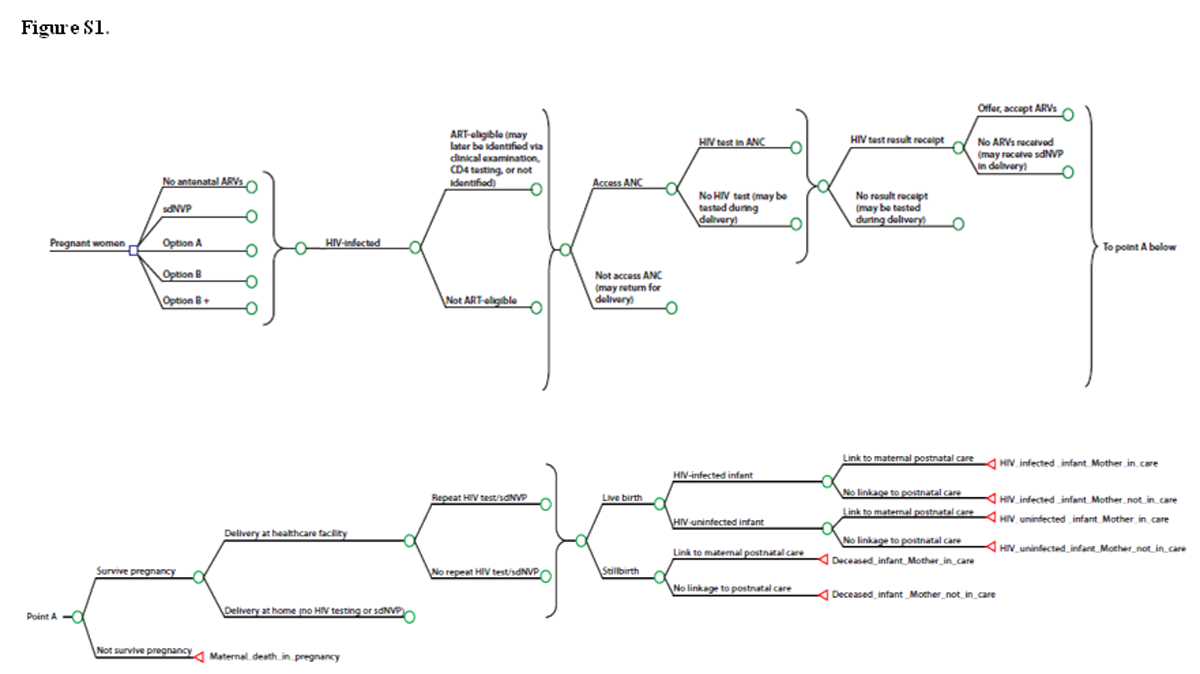

Supplement: S1 Fig — The MTCT model is a decision tree, coded in TreeAgePro software. Pregnant women enter the model at conception. Five possible PMTCT strategies are shown at the decision node, indicated by a square. This analysis examined only Option A. Circles indicate chance nodes, at which events occur based on probabilities derived from published literature. Triangles indicate terminal nodes, representing the clinical outcome of any single pathway through the model. Brackets reflect that the subsequent events emerging to the right of the bracket may follow any of the prior chance nodes included to the left of the bracket. At each chance node, the probabilities of all subsequent modeled events may depend on the PMTCT strategy being simulated and on the prior events leading to that node. For each modeled PMTCT strategy, the series of events shown in the figure may occur. For example, HIV-infected women may be ART-eligible (CD4≤350/μL or WHO Stage 3–4 disease) or non-ART-eligible; ART-eligibility may be identified by CD4 testing, identified by clinical evaluation, or not identified. All women may access ANC, undergo HIV testing in ANC, and receive HIV test results, or may fail to access these steps in the cascade. If identified as HIV-infected, women may be offered ARVs for PMTCT according to the PMTCT strategy being simulated, as well as ART if identified as ART-eligible (not shown). In the base case, women were assumed to receive all ANC services; service uptake was varied in sensitivity analyses. Probabilities for surviving pregnancy depend on receipt of ART; if maternal death occurs, infant death also occurs. Women who survive pregnancy may deliver at a healthcare facility or at home; if they deliver in a healthcare facility, they may access HIV testing (if previous status was unknown or negative), and if identified as HIV-infected at that time, may receive sdNVP in labor (sdNVP was excluded for this analysis). All women surviving pregnancy then experience probabilities of live b [file pone.0117751.s002.tiff]

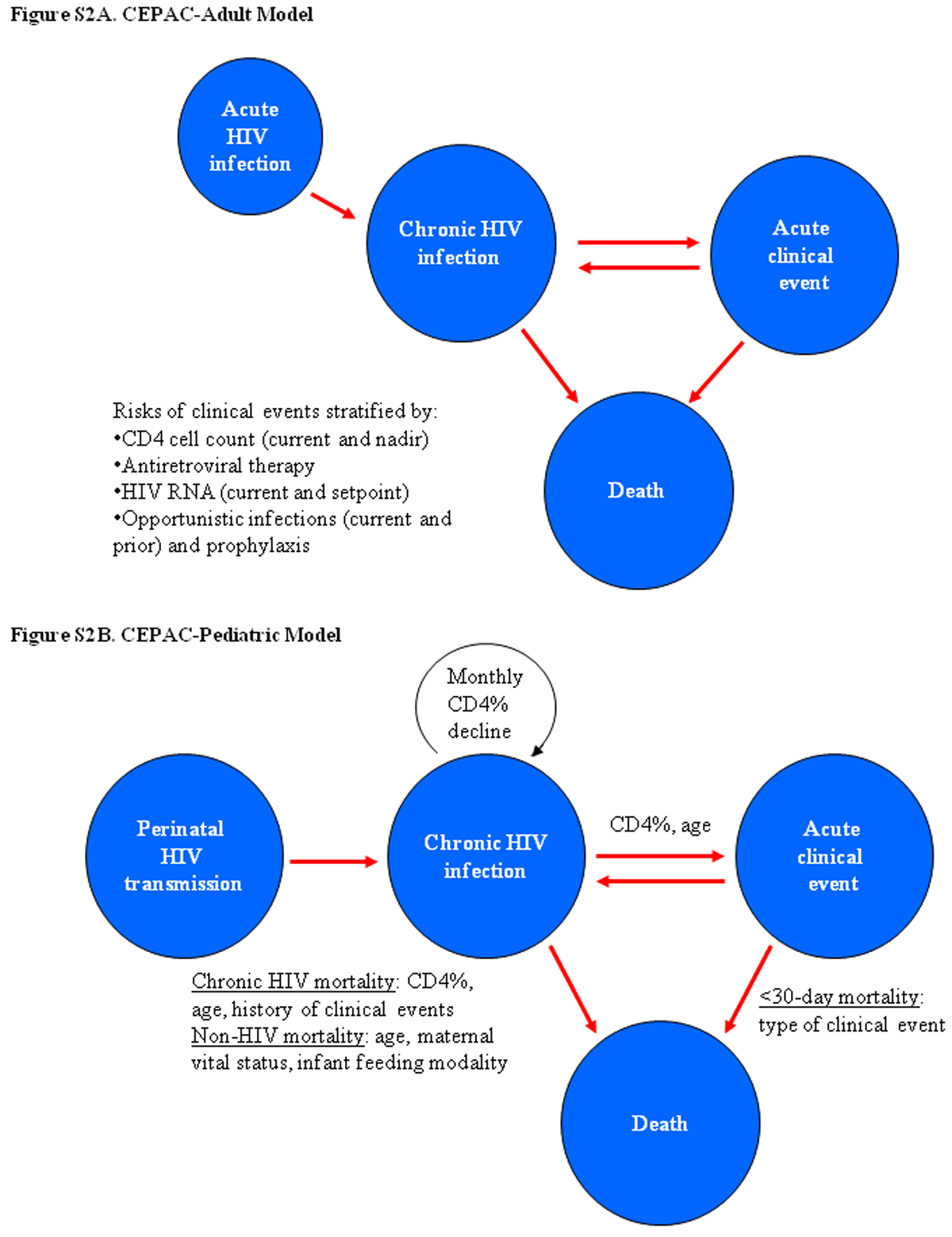

Supplement: S2 Fig — Schematic representations of the adult and infant CEPAC model structures. Women enter the adult model (S2A Fig.) after delivery; for this analysis, all modeled women enter with chronic HIV infection. They then face monthly risks of clinical events including opportunistic infections, medication toxicities, and death; these risks are stratified by the parameters listed in the figure. Life months accrued between presentation to antenatal care and delivery are added to the CEPAC model projections. Infants enter the pediatric model (S2B Fig.) after delivery; for this analysis, all modeled infants enter either HIV-negative or with an intrauterine/intrapartum HIV infection. Infants HIV-negative upon entering the model can either develop a postpartum HIV infection via breastfeeding or remain HIV-negative. HIV-infected infants face monthly risks of clinical events including opportunistic infections, medication toxicities, and death; these risks are stratified by age and CD4%. (TIFF) [file pone.0117751.s003.tiff]
