# Supplementary material for: Point-of-Care CD4 Testing to Inform Selection of Antiretroviral Medications in South African Antenatal Clinics: A Cost-Effectiveness Analysis
Source: PLoS One. 2015 Mar 10;10(3):e0117751. doi: 10.1371/journal.pone.0117751 (PMC4355621; doi:10.1371/journal.pone.0117751)
Supplement: S1 Table — (DOCX) [file pone.0117751.s004.docx]

**Table S1. Complete input parameters for a model of mother-to-child transmission in South Africa (includes parameters listed in manuscript Table 1)**

| **I. Clinical model input parameters** | | | | | | | |
| --- | --- | --- | --- | --- | --- | --- | --- |
| **Ia. Baseline maternal cohort characteristics** | | | | | | | |
| **Variable** | | | | **Base Case Value** | | **Data sources** | |
| Age (mean (SD), years) | | | | 26 (5) | | ^4^ | |
| Mortality during pregnancy | | | | 0.26% | | ^64^ | |
| Proportion ART-eligible^a^ | | | | 44% | | ^4^ | |
| Distribution of initial HIV RNA (% total) | | | |  | | Cape Town AIDS Cohort ^65^ | |
| >100,000 copies/ml | | | | 42 | |  |  |
| 30,001-100,000 copies/ml | | | | 28 | |  |  |
| 10,001-30,000 copies/ml | | | | 18 | |  |  |
| 3,001-10,000 copies/ml | | | | 8 | |  |  |
| 501-3,000 copies/ml | | | | 2 | |  |  |
| ≤ 500 copies/ml | | | | 1 | |  |  |
| **Ib. Uptake of PMTCT services and postnatal care** | | | | | | | |
|  | **CD4 tested**  **(of HIV+)** | **Receiving results**  **(of CD4 tested)** | | | **CD4 tested and receiving results (of HIV+)** | | **Data Sources** |
| Laboratory CD4 testing (base-case) | 96.0% | 86.5% | | | 83.0% | | ^6^ |
| Laboratory CD4 testing (low-access) | 30.0% | 50.0% | | | 15.0% | | ^66^ |
| POC CD4 testing | 99.0% | 95.0% | | | 94.1% | | ^6^ |
|  | | | **Base Case Value (Range)** | | | | **Data sources** |
| Sensitivity of *laboratory* CD4 test for CD4 ≤350/µL^a^ | | | 100% | | | | Assumption |
| Specificity of *laboratory* CD4 test for CD4 ≤350/µL^a^ | | | 100% | | | | Assumption |
| Sensitivity of *POC* CD4 test for CD4 ≤350/µL^a^ | | | 93% (50-100%) | | | | ^67^ |
| Specificity of *POC* CD4 test for CD4 ≤350/µL^a^ | | | 86% (50-100%) | | | |  |
| Duration of breastfeeding (months) | | | 6 (sensitivity analysis: 12) | | | | Assumption |
| Probability of linking to postnatal maternal HIV-related care for women enrolled in antenatal care | | | 100% (guideline-line concordant care) | | | | Assumption |
| Loss to follow-up from postnatal maternal care | | | 0%/year  (sensitivity analyses: 1.4%/year; 9.9%/year) | | | | ^63^ |
| Loss to follow-up from pediatric HIV care | | | 0%/year  (sensitivity analyses: 0.4%/mth; 0.8%/mth) | | | | ^10^ |

**Table S1, continued.**

| **Ic. Mother-to-child transmission risks** | | | | | |
| --- | --- | --- | --- | --- | --- |
| **Maternal HIV status** | **PMTCT regimen received** | | | | |
| **Intrauterine/intrapartum period (one-time risks): base case value (range for sensitivity analyses)** | | | | | |
|  | | **Antenatal AZT^b^** | **Antenatal three-drug ARV regimen** | | **Data sources** |
| CD4 ≤350/µL at conception | | 0.136 (0.068-0.272) | 0.033 (0.017-0.066) | | ^22,45-51^ |
| CD4 >350/µL at conception | | 0.036 (0.018-0.072) | 0.01 (0.005-0.02) | |  |
| **Maternal HIV status** | | **PMTCT regimen received** | | | |
| **Postnatal period (rate/100 person-years, among infants HIV-uninfected at 4-6 weeks of age): base case value (range for sensitivity analyses)** | | | | | |
|  | | **Extended infant NVP** | **Antenatal three-drug ARV regimen** | | **Data sources** |
| CD4 ≤350/µL | | n/a | 4.0 (2.0-8.0) | | ^30,46,48-55^ |
| CD4 >350/µL | | 2.7 (1.4-5.4) | 2.2 (1.1-4.4) | |  |
| **Id. Pediatric disease progression parameters** | | | |  |  |
| **Variable** | | | | **Value** | **Data sources** |
| Probability of live birth | | | | 99.8% | ^64^ |
| **Natural history (in absence of ART)** | | | |  |  |
| Mean monthly decrease in CD4 | | | | 4.0% (months 0-2 if IU/IP infection)  0.5% (all others) | ^7^ |
| Monthly risk of HIV-related death (%, range by CD4 %); <5 years of age | | | |  | |
| No history of OIs | | | | 0.3%-6.3% | ^7^ |
| With history of OIs | | | | 0.3%-24.2% | ^7^ |
| Monthly risk of HIV-related death (%, range by CD4 %); 5-13 years of age | | | |  | |
| No history of OIs | | | | 0.3%-0.6% | ^7^ |
| With history of OIs | | | | 0.3%-2.4% | ^7^ |
| Monthly non-AIDS death probability (%, range by CD4 %) | | | |  |  |
| HIV-infected | | | | 0.04%-0.51% | ^24^ |
| HIV-exposed, uninfected | | | | 0.04%-1.27% |  |
| **Impact of antiretroviral therapy** | | | |  |  |
| Efficacy (% HIV RNA suppression at 24 weeks) | | | |  |  |
| 1^st^-line ART (ABC/3TC/LPV/r) | | | | 91% | ^68-69^ |
| 2^nd^ line ART (AZT/3TC/NVP) | | | | 75% |  |

**Table S1, continued.**

| **Ie. Maternal disease progression parameters** | **Value** | **Data sources** |
| --- | --- | --- |
| **Variable** | **Value** | **Data sources** |
| **Natural history (in absence of antiretroviral therapy)** |  |  |
| Mean monthly decrease in CD4/µL by HIV RNA |  |  |
| >30,000 copies/ml | 6.4 | Multicenter AIDS Cohort Study^70^ |
| 10,001-30,000 copies/ml | 5.4 |  |
| 3,001-10,000 copies/ml | 4.6 |  |
| 501-3,000 copies/ml | 3.7 |  |
| 0-500 copies/ml | 3.0 |  |
| Monthly risk of severe opportunistic infections (%, range by CD4) | |  |
| WHO stage 3-4 |  | Cape Town AIDS Cohort^65^ |
| Visceral | 0.00-1.52 |  |
| Non-visceral | 0.03-2.26 |  |
| Non-specific | 0.00-0.71 |  |
| Bacterial infection | 0.04-0.71 |  |
| Tuberculosis | 0.16-1.96 |  |
| Other severe infection | 0.20-1.67 |  |
| Monthly risk of other clinical conditions (%, range by CD4) | |  |
| Mild fungal infection | 1.76-3.14 | Cape Town AIDS Cohort^65^ |
| Other mild infection | 2.33-2.67 |  |
| Monthly risk of death from severe opportunistic infection (%) |  |  |
| WHO stage 3-4 |  | Cape Town AIDS Cohort^71^ |
| Visceral | 9.21 |  |
| Non-visceral | 2.38 |  |
| Non-specific | 20.00 |  |
| Bacterial infection | 2.94 |  |
| Tuberculosis | 1.82 |  |
| Other severe infection | 6.67 |  |
| Monthly risk of death from other clinical conditions (%) |  |  |
| Mild fungal infection | 0.54 | Cape Town AIDS Cohort^71^ |
| Other mild infection | 0.39 |  |
| Monthly risk of HIV-related death (%, range by CD4) |  |  |
| No history of opportunistic infection | 0.11-4.02 | Cape Town AIDS Cohort^65^ |
| With history of opportunistic infection | 0.11-9.53 |  |
| Relative risk reduction on any ART regimen (%, range by CD4) |  |  |
| HIV-related death | 55-96 | Cotrimo-CI, ANRS 1203^19^ |
| Acute opportunistic infections | 0-32 |  |

**Table S1, continued.**

| **Ie. Maternal disease progression parameters, continued** | **Value** | **Data sources** | |
| --- | --- | --- | --- |
| **Impact of trimethoprim-sulfamethoxazole prophylaxis** |  |  | |
| % Reduction in probability of infection |  |  | |
| Mild fungal infection | 46.37 | Cotrimo-CI^72^ | |
| Mild bacterial diseases | 48.79 |  |  |
| Invasive bacterial diseases | 49.81 |  |  |
| WHO stage 3-4 visceral diseases | 17.86 |  |  |
| Other severe events | 17.88 |  |  |
| Toxicity of trimethoprim-sulfamethoxazole (%, one-time risk) |  |  | |
| Minor toxicity | 18.24 | Cotrimo-CI^61,72^ | |
| Major toxicity | 6.72 |  |  |
| **Impact of antiretroviral therapy** | |  | |
| Efficacy (% HIV RNA suppression at 24 weeks) | |  | |
| 1^st^-line ART (TDF/FTC /EFV) | 90% | OCTANE trial ^73-76^ | |
| 2^nd^ line ART (AZT/3TC/LPV/r) | 72% | ^74-77^ | |
| **II. Economic model input parameters: base case (all costs halved and doubled)** | | | |
| **IIa. Laboratory and medication costs** | **2013 USD** | | **Data sources** |
| CD4 assay (performed once in ANC) | Lab: $14.00 | | ^60^  ^78^ |
|  | POC: $26.00 ($26.00-$100.00) | |  |
| CD4 result return (provider time to receive file and give result) | Lab: $1.00 | | Assumption (nurse time x salary)^58^ |
|  | POC: $0.00 | |  |
| HIV RNA (viral load) test | $56.55 | | ^60^ |
| Antenatal AZT^c^ | $22.62 | | ^59^ (base-case: 13 weeks antenatal drug) |
| Antenatal TDF/3TC/EFV^c^ | Lab: $36.30 | | ^59^ (base-case: *lab*-10 wks; *POC*-13 wks) |
|  | POC: $40.40 | |  |
| Postnatal maternal ART |  | |  |
| 1st-line (TDF/FTC/EFV) | $13.47/month | | ^59^ |
| 2nd line (AZT/3TC/LPV/r) | $40.91/month | | ^59^ |
| Pediatric ART (range by age and weight) |  | |  |
| 1st-line (ABC/3TC/ LPV/r) | $24.71-$40.72/month | | ^25,59^ |
| 2nd line (AZT/3TC/NVP/) | $6.21-$15.23/month | |  |

**Table S1, continued.**

| **II. Economic model input parameters, continued: base case (all costs halved and doubled)** | | | | | |
| --- | --- | --- | --- | --- | --- |
| **IIb. Antenatal care utilization and costs** | | **2013 USD** | | **Notes and data sources** | |
| Routine antenatal care (4 visits) | | $200.00 | | Assumption | |
| HIV testing in antenatal care | |  | |  | |
| Test kit | | $1.45 | | Kit cost: average of ^79-80^; 1.16 kits/person | |
| 10 minutes nurse time (pretest counseling) | | $1.22 | | Assumption (nurse time x salary)^58^ | |
| 25 minutes nurse time (posttest counseling, positive result) | | $3.05 | | Assumption (nurse time x salary)^58^ | |
| 15 minutes nurse time (posttest counseling, negative result) | | $1.83 | | Assumption (nurse time x salary)^58^ | |
| Delivery costs (healthcare facility) | | $60.91 | | ^81^ | |
| **IIc. Pediatric healthcare utilization and costs** |  | |  |  |  |
| **Urgent health care costs** | **Total cost/event** | | | **Data sources** |  |
| Care for acute OI (per event): |  | | |  |  |
| WHO stage 3 | $1,237.63 | | | ^60,62^ |  |
| WHO stage 4 | $2,174.64 | | |  |  |
| Tuberculosis | $1,650.21 | | |  |  |
| **Routine Care Costs for HIV+ children (per month)** | **# Inpatient days** | | **# Outpatient visits** | **Total cost ($)** | **Data sources** |
| CD4 >35% | 0.03 | | 0.30 | 21.39 | Resource utilization:^71^, costs: see Methods above |
| CD4 25-35% | 0.06 | | 0.27 | 28.28 |  |
| CD4 15-25% | 0.08 | | 0.29 | 33.93 |  |
| CD4 5-15% | 0.22 | | 0.29 | 73.54 |  |
| CD4 < 5% | 0.56 | | 0.52 | 166.65 |  |
| **Terminal care, last month of life** | 2.39 | | 0.77 | 653.49 |  |
| **IId. Maternal healthcare utilization and costs** | **# Inpatient days** | | **# Outpatient visits** | **Total cost/event ($)** | **Data sources** |
| **Urgent health care costs (per event, range by OI type)** |  | |  |  | Resource utilization:^71^, costs: see Methods above |
| Care for acute OI (per event): |  | |  |  |  |
| WHO stage 3-4 |  | |  |  |  |
| Visceral | 2.9 | | 3.4 | 874.92 |  |
| Non-visceral | 1.8 | | 2.7 | 560.60 |  |
| Non-specific | 1.3 | | 2.9 | 462.82 |  |
| Bacterial infection | 2.8 | | 2.4 | 822.89 |  |
| Mild fungal infection | 1.2 | | 2.3 | 385.77 |  |
| Tuberculosis | 2.9 | | 2.2 | 827.18 |  |
| Other mild infection | 0.7 | | 2.2 | 260.47 |  |
| Other severe infection | 1.8 | | 2.6 | 557.96 |  |
| **Terminal care, last month of life** | 2.39 | | 0.77 | 653.49 |  |

**Table S1, continued.**

| **II. Economic model input parameters, continued (all costs halved and doubled)** | | | | |
| --- | --- | --- | --- | --- |
| **IId. Maternal healthcare utilization and costs, continued** | **# Inpatient days** | **# Outpatient visits** | **Total cost ($)** | **Data sources** |
| **Routine Care Costs (per month)** |  |  |  |  |
| CD4 >500/µL | 0.03 | 0.30 | 21.39 | Resource utilization: ^71^, costs: see Methods above |
| CD4 351-500/µL | 0.06 | 0.27 | 28.28 |  |
| CD4 201-350/µL | 0.08 | 0.29 | 33.93 |  |
| CD4 51-200/µL | 0.22 | 0.29 | 73.54 |  |
| CD4 <50/µL | 0.56 | 0.52 | 166.65 |  |

**SD**: Standard deviation; **ART**: antiretroviral therapy; **POC**: point-of-care; **PMTCT**: prevention of mother-to-child HIV transmission; **AZT**: azidothymidine; **ARV**: antiretroviral; **NVP**: nevirapine; **OI:** opportunistic infection; **IU:** intrauterine; **IP:** intrapartum; **PP:** postpartum; **ABC:** abacavir; **3TC**: lamivudine; **LPV/r**: lopinavir/ritonavir; **TDF**: tenofovir; **FTC**: emtricabine; **EFV**: efavirenz; **WHO**: World Health Organization.

**a.** Sensitivity and specificity were modeled with regard to true CD4 value of ≤350/µL (sensitivity: assay reports CD4 ≤350/µL when true CD4 is ≤350/µL; specificity: assay reports CD4 >350/µL when true CD4 is >350/µL).

**b. Antenatal AZT:** This reflects the antenatal regimen for women who do not test eligible for ART as per the WHO 2010 PMTCT guidelines.

**c.** In the base-case analysis, 13 weeks of antentatal AZT for non-ART eligible women are assumed in both scenarios, based on median gestational age at booking in South Africa of 26 weeks. For ART-eligible women, 13 weeks of ART are assumed in the *POC* scenario and 3 weeks of AZT and 10 weeks of ART are assumed in the *laboratory* scenario.
