# Supplementary material for: Point-of-Care CD4 Testing to Inform Selection of Antiretroviral Medications in South African Antenatal Clinics: A Cost-Effectiveness Analysis
Source: PLoS One. 2015 Mar 10;10(3):e0117751. doi: 10.1371/journal.pone.0117751 (PMC4355621; doi:10.1371/journal.pone.0117751)
Supplement: S2 Table — (DOCX) [file pone.0117751.s005.docx]

**Table S2. Intermediate (CEPAC model) results ^a^**

| **I. Maternal outcomes (Selected CEPAC-Adult model results)** | | | | | |
| --- | --- | --- | --- | --- | --- |
| **Maternal HIV status and HIV-related care at 6 weeks postpartum** | | **Life expectancy (undiscounted years, from delivery)** | | **Lifetime per-person HIV-related costs (undiscounted, 2013 USD)** | |
| **CD4 ≤350/µL^b^** | |  | |  | |
| On ART in pregnancy, continue lifelong | | 20.22 | | 24,470 | |
| Not on ART in pregnancy, link to postnatal HIV care^c^ (and begin ART during BF) | | 20.18 | | 24,420 | |
| Not on ART in pregnancy, not in postnatal HIV care^c^ | | 11.33 | | 15,150 | |
| **CD4 >350/µL^b^** | |  | |  | |
| On ART in pregnancy, continue lifelong | | 23.35 | | 26,120 | |
| Not on ART in pregnancy or BF, in postnatal HIV care | | 21.54 | | 23,500 | |
| Not in postnatal HIV care^c^ | | 14.63 | | 16,450 | |
| **II. Pediatric outcomes (Selected CEPAC-Pediatric model results)** | | | | | |
| **Infant HIV status and HIV-related care outcomes**  **with 6 months of breastfeeding** | **Life Expectancy (years)** | | **Lifetime**  **Costs ($)** | | **2 Year**  **Survival (%)** |
| **100% of Infants HIV-infected IU-IP, link to care** | | | | |  |
| Discounted | 9.66 | | 9,490 | | 67.7 |
| Undiscounted | 14.00 | | 13,960 | |  |
| **100% of Infants HIV-infected IU-IP, not in care** | | | | |  |
| Discounted | 6.90 | | 8,020 | | 51.8 |
| Undiscounted | 9.98 | | 11,210 | |  |
| **100% of Infants HIV-exposed, uninfected at birth** |  | | | |  |
| Discounted | 24.17 | | 155 | | 90.3 |
| Undiscounted | 54.98 | | 230 | |  |

**BF:** breastfeeding; **IU-IP:** intrauterine-intrapartum; **PP:** postpartum

**a.** CEPAC model outputs were intermediate results, used as inputs to the MTCT model.

**b.** ART eligibility was defined as CD4 ≤350/µL, or WHO stage 3-4 disease.^21,82^

**c.** In the base-case analyses, women and infants were assumed to receive guideline-concordant antenatal care, and to link to postnatal care (defined as enrollment in HIV-related care by 6 weeks postpartum). Results are also shown for women and infants not in care during pregnancy and the postpartum period, to permit sensitivity analyses on the rates of PMTCT service uptake and loss to follow-up. These women and children are assumed to present to HIV care only after the development of a severe opportunistic infection.
