# Supplementary material for: Point-of-Care CD4 Testing to Inform Selection of Antiretroviral Medications in South African Antenatal Clinics: A Cost-Effectiveness Analysis
Source: PLoS One. 2015 Mar 10;10(3):e0117751. doi: 10.1371/journal.pone.0117751 (PMC4355621; doi:10.1371/journal.pone.0117751)
Supplement: S3 Table — (DOCX) [file pone.0117751.s006.docx]

**Table S3. Sensitivity analyses for the comparison of *POC* and *laboratory* CD4 testing in antenatal care**

| **CD4 testing strategy** | **MTCT**  **(6 months)** | **ANC Costs($)** | **Pediatric** | | **Maternal** | | **Total LE undisc**  **(years)** | **Total LE disc**  **(years)** | **Total cost**  **Disc**  **($)** | **Summary** |
| --- | --- | --- | --- | --- | --- | --- | --- | --- | --- | --- |
|  |  |  | **LE undisc**  **(years)** | **LT cost disc^a^**  **($)** | **LE undisc**  **(years)** | **LT cost disc^a^**  **($)** |  |  |  |  |
| *Laboratory* CD4 uptake (% receiving *lab* CD4 test * % receiving result) | | | | | | | | | | |
| *Laboratory uptake=83% (base case)* | 5.7% | 310 | 53.18 | 520 | 21.15 | 15,440 | 74.33 | 38.28 | 16,270 | *POC* more effective, less expensive than *laboratory* |
| *Laboratory uptake=15% (low laboratory access scenario)* | 8.7% | 295 | 51.93 | 810 | 21.14 | 15,430 | 73.07 | 37.82 | 16,540 | *POC* more effective, less expensive than *laboratory* |
| *POC* | 5.3% | 325 | 53.35 | 480 | 21.15 | 15,440 | 74.50 | 38.34 | 16,250 | -- |
| *POC* assay cost | | | | | | | | | | |
| *Laboratory* | 5.7% | 310 | 53.18 | 520 | 21.15 | 15,440 | 74.33 | 38.28 | 16,270 | -- |
| *POC assay cost = $26 (base case)* | 5.3% | 325 | 53.35 | 480 | 21.15 | 15,440 | 74.50 | 38.34 | 16,250 | *POC* more effective, less expensive than *laboratory* |
| *POC assay cost = $51* | 5.3% | 350 | 53.35 | 480 | 21.15 | 15,440 | 74.50 | 38.34 | 16,270 | *POC* more effective, equally expensive than *laboratory* |
| *POC* CD4 uptake (% receiving *POC* CD4 test * % receiving result) | | | | | | | | | | |
| *Laboratory* | 5.7% | 310 | 53.18 | 520 | 21.15 | 15,440 | 74.33 | 38.28 | 16,270 | -- |
| *POC uptake = 90.3% (base case)* | 5.3% | 325 | 53.35 | 480 | 21.15 | 15,440 | 74.50 | 38.34 | 16,250 | *POC* more effective, less expensive than *laboratory* |
| *POC uptake=89%* | 5.5% | 325 | 53.25 | 500 | 21.15 | 15,440 | 74.40 | 38.31 | 16,270 | *POC* more effective, equally expensive than *laboratory* |
| *POC uptake=85%* | 5.7% | 325 | 53.18 | 520 | 21.15 | 15,440 | 74.33 | 38.28 | 16,290 | *POC* equally effective, more expensive than  *laboratory* |
| *POC* assay sensitivity and specificity | | | | | | | | | | |
| *Laboratory* | 5.7% | 310 | 53.18 | 520 | 21.15 | 15,440 | 74.33 | 38.28 | 16,270 | -- |
| *POC test sensitivity = 93%, specificity = 86% (base case)* | 5.3% | 325 | 53.35 | 480 | 21.15 | 15,440 | 74.50 | 38.34 | 16,250 | *POC* more effective, less expensive than *laboratory* |
| *POC test sensitivity=89%, specificity = 86%* | 5.5% | 325 | 53.28 | 500 | 21.15 | 15,440 | 74.43 | 38.31 | 16,270 | *POC* more effective, equally expensive than *laboratory* |
| *POC test sensitivity=84%, specificity = 86%* | 5.7% | 325 | 53.19 | 520 | 21.15 | 15,440 | 74.34 | 38.28 | 16,290 | *POC* equally effective (discounted), more expensive than *laboratory* |
| *POC test sensitivity=93%, specificity = 100%* | 5.5% | 325 | 53.26 | 500 | 21.15 | 15,440 | 74.41 | 38.31 | 16,270 | *POC* more effective, equally expensive than *laboratory* |
| *POC test sensitivity=93%, specificity = 80%* | 5.2% | 325 | 53.38 | 470 | 21.15 | 15,440 | 74.53 | 38.35 | 16,240 | *POC* more effective, less expensive than *laboratory* |
| *POC test sensitivity=93%, specificity = 50%* | 4.8% | 325 | 53.55 | 430 | 21.15 | 15,440 | 74.70 | 38.41 | 16,200 | *POC* more effective, less expensive than *laboratory* |

**Table S3, continued.**

| **CD4 testing strategy** | **MTCT**  **(6 months)** | **ANC Costs**  **($)** | **Pediatric** | | **Maternal** | | **Total LE undisc**  **(years)** | **Total LE disc**  **(years)** | **Total cost disc**  **($)** | **ICER** |
| --- | --- | --- | --- | --- | --- | --- | --- | --- | --- | --- |
|  |  |  | **LE undisc**  **(years)** | **LT cost disc^a^**  **($)** | **LE undisc**  **(years)** | **LT cost disc^a^**  **($)** |  |  |  |  |
| Antenatal loss to follow-up = 25%^b^ | | | | | | | | | | |
| *Laboratory* | 10.0% | 310 | 51.40 | 930 | 21.15 | 15,440 | 72.55 | 37.68 | 16,370 | *POC* more effective, less expensive than *laboratory* |
| *POC* | 9.7% | 325 | 51.51 | 900 | 21.15 | 15,440 | 72.66 | 38.28 | 16,340 |  |
| Antenatal loss to follow-up = 50%^b^ | | | | | | | | | | |
| *Laboratory* | 14.3% | 310 | 49.60 | 1340 | 21.14 | 15,440 | 70.74 | 37.00 | 16,780 | *POC* more effective, less expensive than *laboratory* |
| *POC* | 14.1% | 325 | 49.67 | 1320 | 21.14 | 15,440 | 70.81 | 37.03 | 16,760 |  |
| Loss to follow-up between delivery and postnatal care = 25%^c^ | | | | | | | | | | |
| *Laboratory* | 6.0% | 310 | 53.06 | 540 | 19.20 | 14,370 | 72.26 | 37.01 | 14,910 | *POC* more effective, less expensive than *laboratory* |
| *POC* | 5.6% | 325 | 53.22 | 500 | 19.20 | 14,370 | 72.42 | 37.07 | 14,870 |  |
| Loss to follow-up between delivery and postnatal care = 50%^c^ | | | | | | | | | | |
| *Laboratory* | 6.2% | 310 | 52.94 | 560 | 17.25 | 13,300 | 70.19 | 35.75 | 13,860 | *POC* more effective, less expensive than *laboratory* |
| *POC* | 5.8% | 325 | 53.10 | 520 | 17.25 | 13,300 | 70.35 | 35.81 | 13,820 |  |
| Maternal loss to follow-up = 0.1187% per month | | | | | | | | | | |
| *Laboratory* | 5.7% | 310 | 53.18 | 520 | 19.29 | 14,060 | 72.47 | 37.31 | 14,890 | *POC* more effective, less expensive than *laboratory* |
| *POC* | 5.3% | 325 | 53.35 | 480 | 19.29 | 14,060 | 72.64 | 37.37 | 14,870 |  |
| Maternal loss to follow-up = 0.8268% per month | | | | | | | | | | |
| *Laboratory* | 5.7% | 310 | 53.18 | 520 | 13.09 | 9,630 | 66.27 | 33.80 | 10,460 | *POC* more effective, less expensive than *laboratory* |
| *POC* | 5.3% | 325 | 53.35 | 480 | 13.09 | 9,630 | 66.44 | 33.86 | 10,440 |  |
| Maternal loss to follow-up = 0.8268% per month pre-ART initiation and 0.1187% per month post-ART initiation | | | | | | | | | | |
| *Laboratory* | 5.7% | 310 | 53.18 | 520 | 18.25 | 13,390 | 71.43 | 36.69 | 14,220 | *POC* more effective, less expensive than *laboratory* |
| *POC* | 5.3% | 325 | 53.35 | 480 | 18.25 | 13,390 | 71.60 | 36.75 | 14,200 |  |
| Pediatric loss to follow-up = 0.4% per month | | | | | | | | | | |
| *Laboratory* | 5.7% | 310 | 53.05 | 450 | 21.15 | 15,440 | 74.20 | 38.21 | 16,200 | *POC* more effective, less expensive than *laboratory* |
| *POC* | 5.3% | 325 | 53.23 | 420 | 21.15 | 15,440 | 74.38 | 38.27 | 16,190 |  |
| Pediatric loss to follow-up = 0.8% per month | | | | | | | | | | |
| *Laboratory* | 5.7% | 310 | 52.98 | 410 | 21.15 | 15,440 | 74.13 | 38.18 | 16,160 | *POC* more effective, less expensive than *laboratory* |
| *POC* | 5.3% | 325 | 53.16 | 380 | 21.15 | 15,440 | 74.31 | 38.24 | 16,150 |  |
| Breastfeeding duration = 12 months (MTCT risks for 12 months shown) | | | | | | | | | | |
| *Laboratory* | 7.1% | 310 | 52.53 | 630 | 21.15 | 15,440 | 73.68 | 38.05 | 16,380 | *POC* more effective, less expensive than *laboratory* |
| *POC* | 6.7% | 325 | 52.70 | 590 | 21.15 | 15,440 | 73.85 | 38.11 | 16,360 |  |

**Table S3, continued.**

|  | |  | |  | **Pediatric** | | | | **Maternal** | | | | **Total LE undisc**  **(years)** | | **Total LE disc**  **(years)** | |  | |  | |
| --- | --- | --- | --- | --- | --- | --- | --- | --- | --- | --- | --- | --- | --- | --- | --- | --- | --- | --- | --- | --- |
| **CD4 testing strategy** | | **MTCT**  **(6 months)** | | **ANC Costs**  **($)** | **LE undisc**  **(years)** | | **LT cost disc^a^**  **($)** | | **LE undisc**  **(years)** | | **LT cost disc^a^**  **($)** | |  |  |  |  | **Total cost**  **($)** | | **ICER** | |
| Healthcare costs doubled | | | | | | | | | | | | | | | | | | | | |
| *Laboratory* | 5.7% | | 570 | | | 53.18 | | 910 | | 21.15 | | 26,690 | | 74.33 | | 38.28 | | 28,170 | | *POC* more effective, less expensive than *laboratory* |
| *POC* | 5.3% | | 585 | | | 53.35 | | 840 | | 21.15 | | 26,690 | | 74.50 | | 38.34 | | 28,120 | |  |
| Healthcare costs halved | | | | | | | | | | | | | | | | | | | | |
| *Laboratory* | 5.7% | | 180 | | | 53.18 | | 290 | | 21.15 | | 9,810 | | 74.33 | | 38.28 | | 10,280 | | *POC* more effective, less expensive than *laboratory* |
| *POC* | 5.3% | | 190 | | | 53.35 | | 270 | | 21.15 | | 9,810 | | 74.50 | | 38.34 | | 10,270 | |  |
| Medication costs doubled | | | | | | | | | | | | | | | | | | | | |
| *Laboratory* | 5.7% | | 335 | | | 53.18 | | 650 | | 21.15 | | 19,580 | | 74.33 | | 38.28 | | 20,570 | | *POC* more effective, less expensive than *laboratory* |
| *POC* | 5.3% | | 350 | | | 53.35 | | 600 | | 21.15 | | 19,580 | | 74.50 | | 38.34 | | 20,530 | |  |
| Medication costs halved | | | | | | | | | | | | | | | | | | | | |
| *Laboratory* | 5.7% | | 295 | | | 53.18 | | 450 | | 21.15 | | 13,370 | | 74.33 | | 38.28 | | 14,120 | | *POC* more effective, less expensive than *laboratory* |
| *POC* | 5.3% | | 310 | | | 53.35 | | 420 | | 21.15 | | 13,370 | | 74.50 | | 38.34 | | 14,100 | |  |
| MTCT risks doubled | | | | | | | | | | | | | | | | | | | | |
| *Laboratory* | | 9.9% | | 310 | | 51.47 | | 910 | | 21.15 | | 15,440 | | 72.62 | | 37.66 | | 16,660 | | *POC* more effective, less expensive than *laboratory* |
| *POC* | | 9.1% | | 325 | | 51.77 | | 840 | | 21.15 | | 15,440 | | 72.92 | | 37.78 | | 16,610 | |  |
| MTCT risks halved | | | | | | | | | | | | | | | | | | | | |
| *Laboratory* | | 3.7% | | 310 | | 54.05 | | 320 | | 21.15 | | 15,440 | | 75.20 | | 38.58 | | 16,070 | | *POC* more effective, equally expensive than *laboratory* |
| *POC* | | 3.3% | | 325 | | 54.13 | | 300 | | 21.15 | | 15,440 | | 75.28 | | 38.62 | | 16,070 | |  |
| Discount rate = 20% | | | | | | | | | | | | | | | | | | | | |
| *Laboratory* | | 5.7% | | 310 | | 53.18 | | 200 | | 21.15 | | 4,220 | | 74.33 | | 10.04 | | 4,730 | | *POC* equally effective (discounted), equally expensive |
| *POC* | | 5.3% | | 325 | | 53.35 | | 180 | | 21.15 | | 4,220 | | 74.50 | | 10.04 | | 4,730 | |  |

**POC:** point-of-care; **ANC**: antenatal care; **MTCT:** mother-to-child HIV transmission; **LE:** life expectancy; **LT**: lifetime;

**ICER**: incremental cost-effectiveness ratio.

**a.** All costs (except ANC costs, because they accrued in year 1) are discounted at 3% annually for cost-effectiveness analyses. All costs are in 2013 USD.

**b**. Women are assigned the cost of medications for PMTCT (leading ANC costs to be equal to the base case), but assumed to be lost to follow-up after filling prescriptions, and do not receive clinical benefits of these medication. To isolate the impact of antenatal loss to follow-up, in these analyses, women and infants are assumed to return to care and receive guideline-concordant care by 6 weeks postpartum.

**c**. Loss to follow-up in these analyses occurs only between delivery and 6 weeks postpartum. Those who are lost to follow-up after delivery are modeled to return to care after development of a severe opportunistic infection. To isolate the impact of immediate postnatal loss to follow-up, after linking to care, women and infants are again assumed to receive guideline-concordant care.
