# Supplementary material for: Point-of-Care CD4 Testing to Inform Selection of Antiretroviral Medications in South African Antenatal Clinics: A Cost-Effectiveness Analysis
Source: PLoS One. 2015 Mar 10;10(3):e0117751. doi: 10.1371/journal.pone.0117751 (PMC4355621; doi:10.1371/journal.pone.0117751)
Supplement: S4 Table — (DOCX) [file pone.0117751.s007.docx]

**Table S4. Cumulative ANC and pediatric costs and pediatric survival over the first five years after delivery (undiscounted; input data for Manuscript Figure 4)**

|  | **Costs (undiscounted, 2013 USD)** | | | **Pediatric survival (%)** |
| --- | --- | --- | --- | --- |
| **Regimen** | **ANC** | **Pediatric** | **Total** |  |
| **6 months after delivery** | | | | |
| *POC* | 325 | 95 | 420 | 93.5 |
| *Laboratory* | 310 | 105 | 415 | 93.5 |
| *"Low lab access"* | 295 | 140 | 435 | 93.0 |
| **12 months after delivery** | | | | |
| *POC* | 325 | 110 | 435 | 91.3 |
| *Laboratory* | 310 | 125 | 435 | 91.2 |
| *"Low lab access"* | 295 | 170 | 465 | 90.6 |
| **18 months after delivery** | | | | |
| *POC* | 325 | 125 | 450 | 89.9 |
| *Laboratory* | 310 | 140 | 450 | 89.8 |
| *"Low lab access"* | 295 | 195 | 490 | 89.2 |
| **24 months after delivery** | | | | |
| *POC* | 325 | 140 | 465 | 89.1 |
| *Laboratory* | 310 | 155 | 465 | 89.0 |
| *"Low lab access"* | 295 | 220 | 515 | 88.3 |
| **36 months after delivery** | | | | |
| *POC* | 325 | 165 | 490 | 88.5 |
| *Laboratory* | 310 | 180 | 490 | 88.3 |
| *"Low lab access"* | 295 | 260 | 555 | 87.6 |
| **48 months after delivery** | | | | |
| *POC* | 325 | 190 | 515 | 87.8 |
| *Laboratory* | 310 | 205 | 515 | 87.7 |
| *"Low lab access"* | 295 | 300 | 595 | 86.8 |
| **60 months after delivery** | | | | |
| *POC* | 325 | 215 | 540 | 87.3 |
| *Laboratory* | 310 | 230 | 540 | 87.1 |
| *"Low lab access"* | 295 | 340 | 635 | 86.2 |

**ANC**: antenatal care; **POC**: point-of-care
